# Task-adaptive multimodal molecular representations for structure-sensitive property prediction

**DOI:** 10.1039/d6sc02982e

**Published:** 2026-09-02

**Authors:** Shaolong Lin, Silong Zhai, Shihang Wang, Xinke Zhan, Yuquan Li, Weihong Li, Li Qin, Lin Shi, Yanan Tian, Kai Xu, Kewei Zhou, Chunbin Gu, Huanxiang Liu, Xiaojun Yao

**Affiliations:** a Faculty of Applied Sciences, Macao Polytechnic University Macao 999078 China hxliu@mpu.edu.mo xjyao@mpu.edu.mo; b Department of Computer Science and Engineering, Chinese University of Hong Kong 999077 Hong Kong gchb4science@gmail.com; c State Key Laboratory of Public Big Data, College of Computer Science and Technology, Guizhou University Guizhou 550025 China

## Abstract

Structure-sensitive properties (SSPs), including activity cliffs and chirality-dependent properties, challenge molecular machine learning because small structural perturbations can cause abrupt property changes and invalidate smooth structure–property assumptions. Here, we present CAMF (Chirality- and Activity-cliff-aware Multimodal Framework), a task-adaptive framework that models SSPs through selective integration of complementary molecular evidence. To systematically evaluate this problem, we construct SSPBench, a benchmark spanning 77 conventional ADMET and physicochemical tasks together with activity-cliff and chirality-sensitive benchmarks. CAMF integrates molecular embeddings and expert-defined descriptors using random-forest-based feature selection and adaptive fusion, enabling property-specific prioritization of informative signals while reducing multimodal redundancy. Across ten baselines, CAMF achieves the best overall performance on SSP tasks, improving mean *R*^2^ by up to 29.5% on activity-cliff datasets and reducing MAE by up to 23.3% on 90 364 chiral molecules with TD-DFT-computed optical rotatory strengths. Ablation analyses show that these gains arise from task-adaptive multimodal integration rather than naive feature concatenation. More broadly, our results reveal that modality relevance is strongly task-dependent, with descriptors and 3D geometry becoming especially important in non-smooth property regimes. Case studies further support the interpretability and practical utility of CAMF in identifying activity-associated substructures and clinically relevant toxicity liabilities.

## Abstract 2

This work establishes SSPBench and presents CAMF for adaptive multimodal representation learning, improving the prediction of activity cliffs, chirality-dependent endpoints, and other structure-sensitive properties.

## Graphical abstract

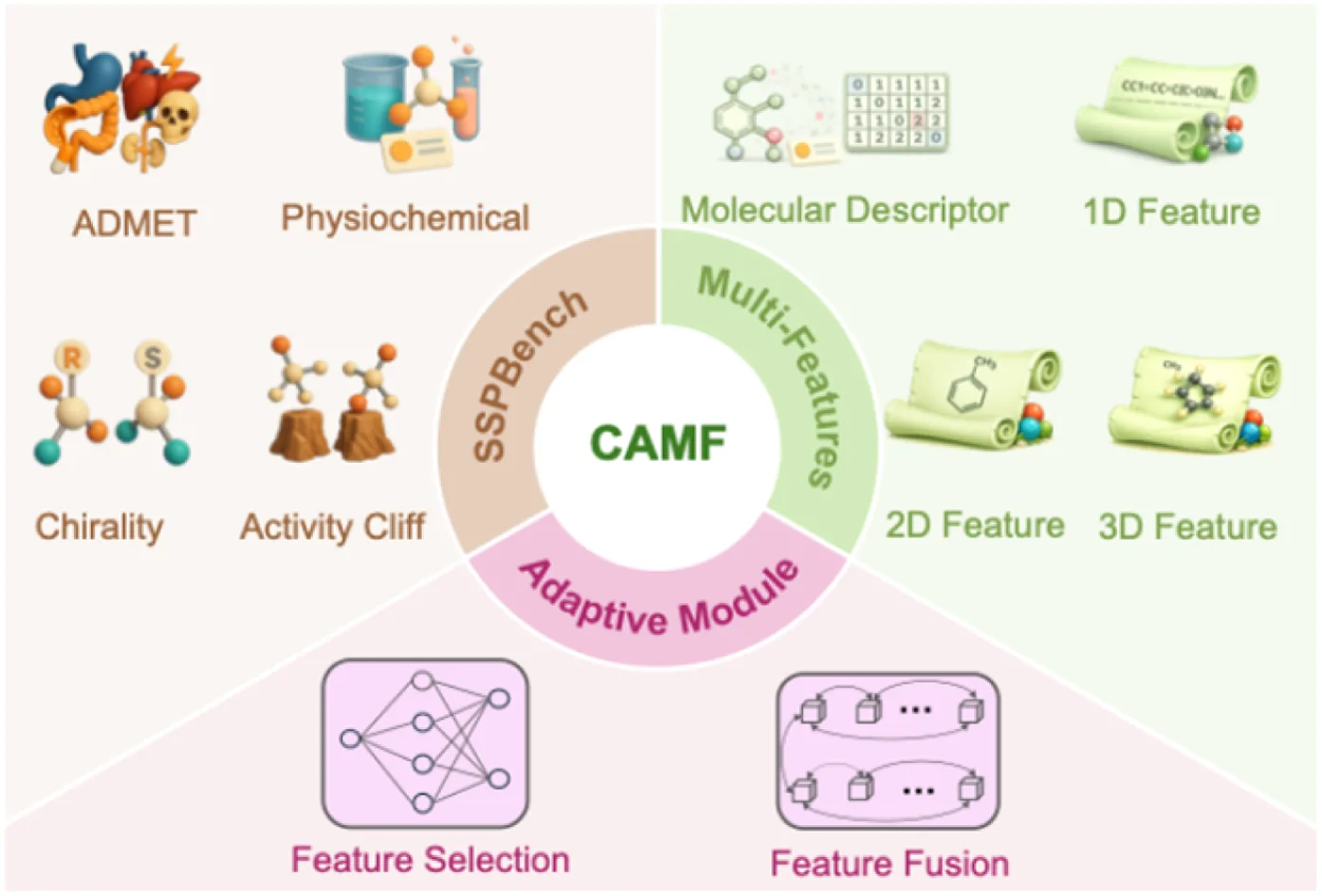

## 1. Introduction

Molecular property prediction (MPP) has become a central task in molecular machine learning because it enables the inference of biochemical and pharmacological behaviors directly from the molecular structure, thereby supporting lead identification, optimization, and safety assessment in drug discovery.^1–4^ Despite substantial progress, reliable prediction remains particularly challenging for structure-sensitive properties (SSPs), in which subtle structural perturbations can induce disproportionately large changes in measured properties.^5,6^ Such non-smooth structure–property relationships are exemplified by activity cliffs, where highly similar compounds display markedly different bioactivities, and by chiral molecules, whose stereoisomers can exhibit distinct biological or physicochemical behaviors.^7,8^ Because these properties are especially relevant to lead optimization and safety evaluation, SSPs represent a stringent and practically important test of molecular representation learning.^9^

A major obstacle arises from how molecules are represented. Most modern molecular representation learning strategies, including self-supervised learning (SSL), are built on an implicit smoothness assumption: structurally similar molecules should occupy nearby positions in representation space and tend to have similar properties.^10,11^ This assumption is often reasonable for conventional physicochemical or ADMET tasks, but it becomes fragile for SSPs, where minute chemical changes may trigger abrupt property shifts. In such cases, structural similarity is no longer a reliable surrogate for property similarity, and representations optimized to preserve global structural resemblance may fail to capture the local, property-determining signals required for accurate prediction.^9^ Indeed, recent studies have shown that current SSL-based molecular representations can underperform even hand-crafted molecular descriptors on challenging MPP tasks.^12^

These observations suggest that the major bottleneck in SSP prediction is not merely insufficient model capacity, but a mismatch between the structural evidence captured by current representations and the signals that actually govern the target property.^9^ For SSPs, no single molecular view may be sufficient because different property regimes can depend on distinct levels of structural information, ranging from topology and local substructures to stereochemical and physicochemical cues.^13^

This motivates the use of multimodal molecular representations, which combine complementary structural views such as molecular graphs, sequence-like representations, and expert descriptors. Although multimodal integration has improved performance on standard molecular benchmarks, its role in SSP prediction remains poorly understood.^14,15^ In particular, it is still unclear which modalities are genuinely informative for different SSP regimes, how their contributions vary across task families, and how multimodal fusion can be designed to avoid redundancy and feature misalignment in high-dimensional representation spaces.

Here, we present CAMF (Chirality- and Activity-cliff-aware Multimodal Framework), an adaptive modality-complementary framework for modeling SSPs. To systematically evaluate representation learning across both conventional and structure-sensitive regimes, we construct SSPBench, a large-scale benchmark spanning routine ADMET and physicochemical tasks together with 30 activity-cliff datasets and a chiral molecular set of 90 364 compounds annotated with TD-DFT-computed optical rotatory strengths. Built on this benchmark, we developed CAMF (Chirality- and Activity-cliff-aware Multimodal Framework), a task-adaptive modality-complementary framework that integrates molecular encoders and descriptors through feature selection and adaptive fusion. Across diverse MPP tasks, CAMF improves both predictive accuracy and generalization and shows particularly strong gains on activity-cliff and chirality-related predictions. Collectively, our results reveal that modality relevance in molecular representation learning is strongly task-dependent, and that SSPs require selective integration of complementary structural evidence rather than generic multimodal aggregation.

## 2. Results

### 2.1. Overview of SSPBench and CAMF

To determine whether modality complementarity is broadly useful or becomes especially important for non-smooth structure–property relationships, we constructed SSPBench, a benchmark spanning both conventional molecular property prediction tasks and structure-sensitive properties (SSPs) (Fig. 1A). SSPBench includes routine ADMET and physicochemical tasks to assess general predictive performance, together with activity-cliff and chirality-related datasets that specifically probe sensitivity to subtle structural, stereochemical, and conformational perturbations. This benchmark design enables us to systematically examine whether different property regimes favor different molecular modalities and whether task-adaptive multimodal integration is particularly beneficial for SSP prediction. The overall benchmark composition is summarized in Fig. S1, and the detailed task and dataset statistics are provided in Tables S1 and S2.

**Fig. 1:**
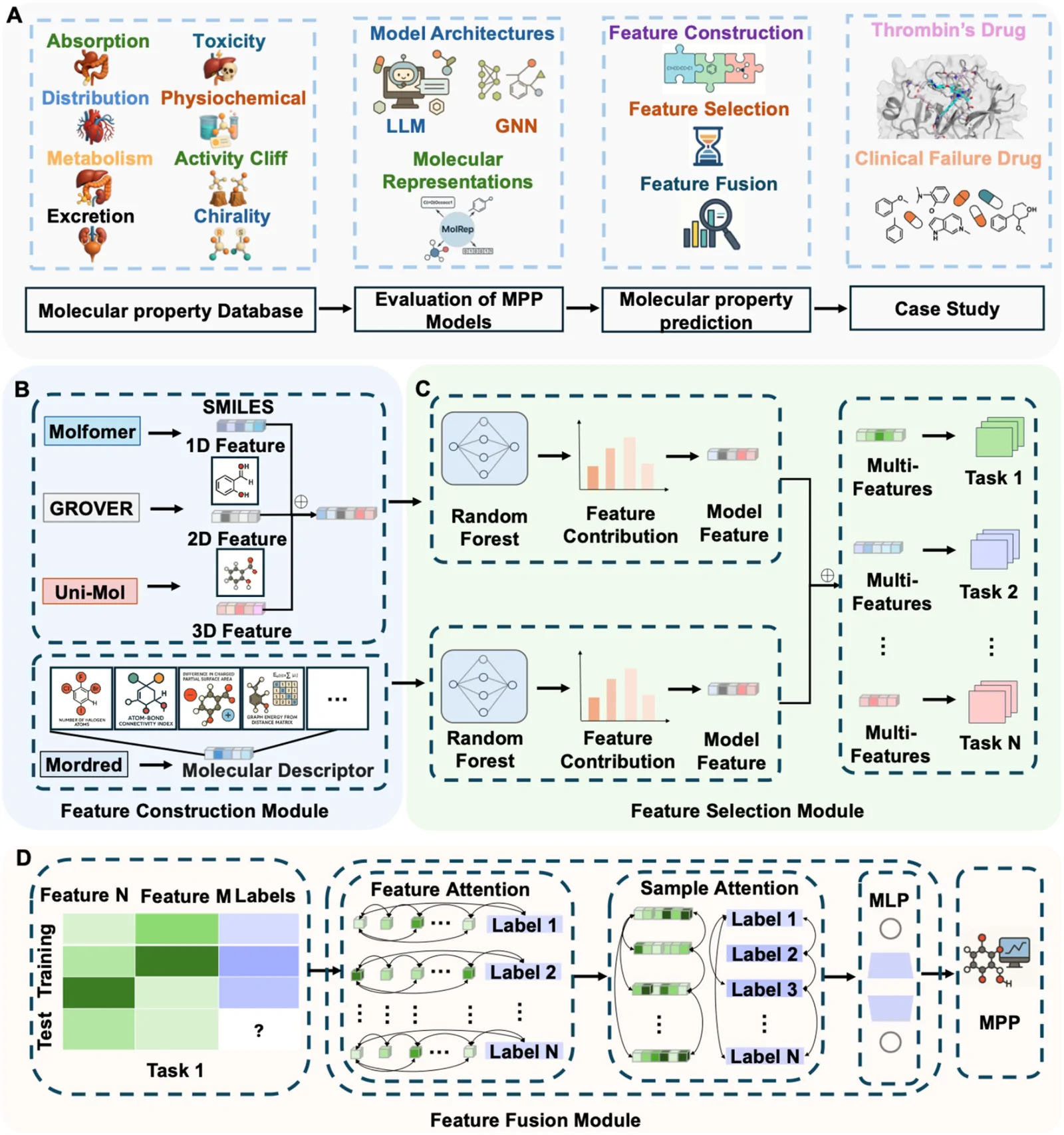
Overview of SSPBench and CAMF. (A) The study comprises four components: construction of SSPBench; large-scale benchmarking of molecular representation models; development of CAMF for task-adaptive multimodal prediction; and representative case studies on activity-related substructures and clinically failed drugs. (B)–(D) Architecture of CAMF. (B) Complementary embedding features and molecular descriptors are generated. (C) Random-forest-based feature selection removes redundancy and identifies task-optimized feature subsets. (D) An attention-based fusion module enhances feature interaction and supports adaptive multimodal prediction and analysis.

Built on SSPBench, we developed CAMF, a task-adaptive modality-complementary framework for molecular property prediction (Fig. 1B–D). CAMF consists of three main components: multimodal feature construction from complementary molecular representations, task-adaptive feature selection to suppress redundancy, and adaptive fusion for property-specific prediction. Based on large-scale benchmarking, we selected strong and complementary encoders from 1D, 2D, and 3D modalities and further incorporated molecular descriptors as expert-defined features. CAMF then combines random-forest-based feature selection with an attention-based fusion module to selectively prioritize property-relevant molecular evidence. In the following sections, we first evaluate CAMF on activity-cliff and chirality-sensitive benchmarks, then analyze modality preferences on conventional tasks, and finally examine its interpretability and practical utility through representative case studies.

### 2.2. CAMF improves prediction on activity-cliff landscapes

Activity cliffs represent a stringent test of molecular representation learning because subtle, localized structural modifications can induce disproportionately large changes in bioactivity, thereby weakening the usual link between molecular similarity and property similarity and making structure–activity relationships markedly non-smooth.^8^ To evaluate whether CAMF can capture such fine-grained perturbations, we assessed its performance on 30 activity-cliff datasets in SSPBench and compared it against ten baseline models, including Chemprop,^16^ AttentiveFP,^17^ FP-GNN,^18^ MolCLR,^19^ MolMCL,^9^ GROVER,^20^ MoLFormer,^21^ Uni-Mol,^22^ KPGT,^14^ and RF.^23^ Model performance was quantified using *R*^2^ as the primary evaluation metric. For clarity, Table 1 reports representative strong baselines, whereas the full comparison against all ten baselines is provided in Fig. 2 and Table S3.

**Table 1:** *R* ^2^ scores of 10 methods across 30 binding-potency prediction tasks using activity-cliff datasets. Details are provided in Table S3

Task	MoLFormer	GROVER	Uni-Mol	CAMF_emb	CAMF_des	CAMF
CHEMBL1862_Ki	0.68	0.68	0.75	0.75	0.75	0.78
CHEMBL1871_Ki	0.57	0.49	0.62	0.59	0.65	**0.66**
CHEMBL2034_Ki	0.44	0.54	0.39	0.41	0.54	**0.56**
CHEMBL204_Ki	0.72	0.56	0.45	0.70	0.75	0.76
CHEMBL2047_EC50	0.47	0.40	0.58	0.48	0.56	0.57
CHEMBL214_Ki	0.60	0.49	0.31	0.56	0.61	**0.64**
CHEMBL2147_Ki	0.88	0.75	**0.91**	0.88	0.89	**0.91**
CHEMBL218_EC50	0.51	0.44	0.31	0.45	0.53	0.56
CHEMBL219_Ki	0.46	0.42	0.45	0.44	0.54	**0.61**
CHEMBL228_Ki	0.65	0.50	**0.73**	0.55	0.68	0.68
CHEMBL231_Ki	0.65	0.62	0.46	0.70	0.72	**0.75**
CHEMBL233_Ki	0.61	0.51	0.44	0.58	0.66	0.66
CHEMBL234_Ki	0.58	0.53	0.40	0.56	0.64	0.65
CHEMBL235_EC50	0.57	0.61	0.58	0.61	0.65	**0.66**
CHEMBL236_Ki	0.67	0.58	0.71	0.67	0.71	**0.72**
CHEMBL237_EC50	**0.71**	0.68	0.66	0.63	**0.71**	**0.71**
CHEMBL237_Ki	0.66	0.71	0.73	0.68	0.73	**0.74**
CHEMBL238_Ki	0.65	0.65	**0.74**	0.64	0.72	**0.74**
CHEMBL239_EC50	0.46	0.47	0.53	0.53	0.58	0.57
CHEMBL244_Ki	0.75	0.71	0.71	0.74	0.80	**0.80**
CHEMBL262_Ki	0.49	0.39	**0.59**	0.32	0.50	0.55
CHEMBL264_Ki	0.65	0.51	0.69	0.62	0.68	**0.70**
CHEMBL2835_Ki	0.77	0.73	0.83	0.84	0.83	**0.85**
CHEMBL287_Ki	0.51	0.42	0.60	0.53	0.61	0.64
CHEMBL2971_Ki	0.76	0.72	0.76	0.78	0.78	0.79
CHEMBL3979_EC50	0.61	0.59	0.53	0.59	0.68	0.67
CHEMBL4005_Ki	0.53	0.62	0.44	0.61	0.64	**0.65**
CHEMBL4203_Ki	0.20	0.34	0.30	0.25	0.29	0.31
CHEMBL4616_EC50	0.28	0.31	0.35	0.29	0.37	0.39
CHEMBL4792_Ki	0.53	0.59	0.49	0.60	0.68	**0.69**
Average (*R*^2^)	0.59	0.54	0.57	0.59	0.65	**0.665**

**Fig. 2:**
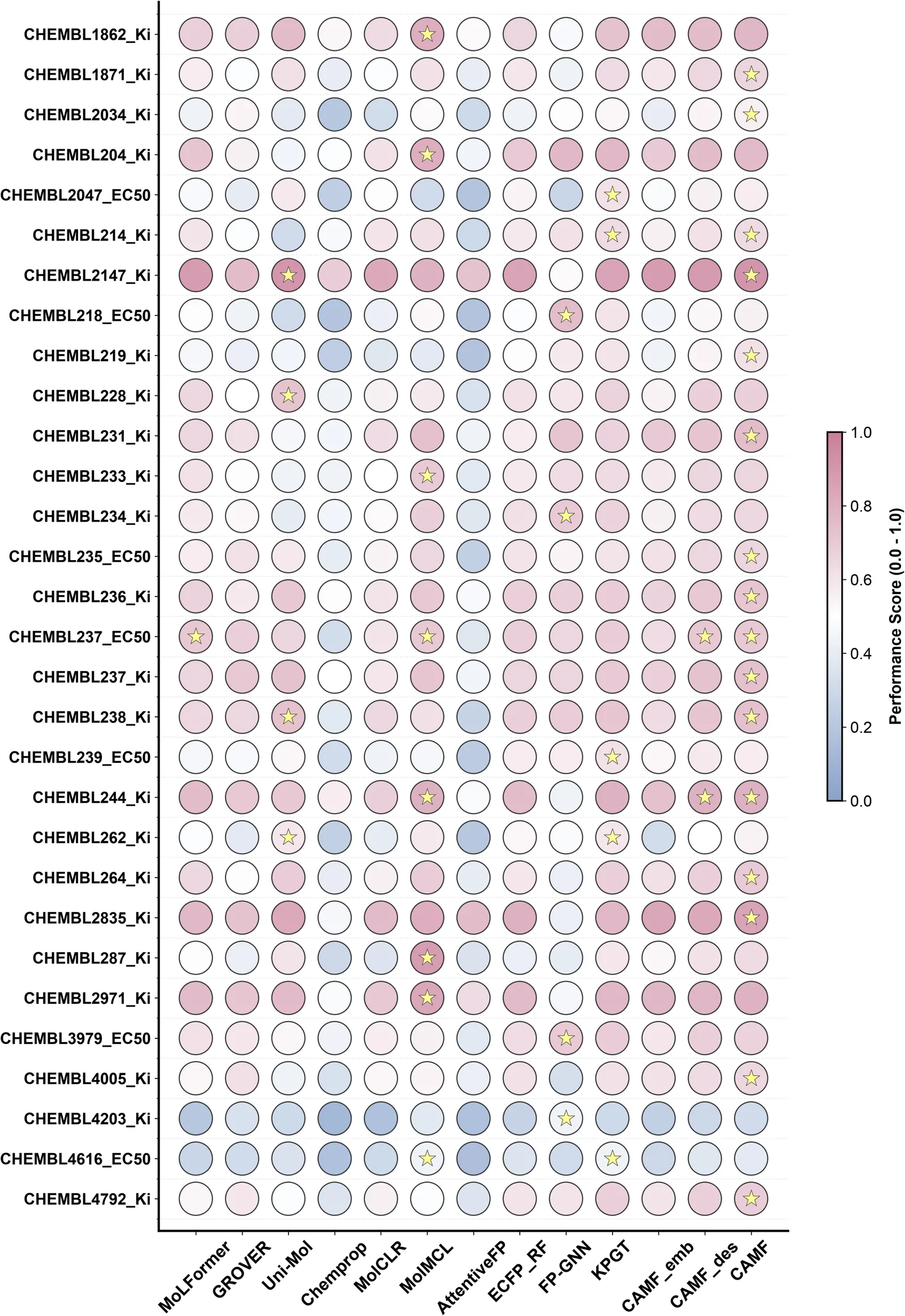
Benchmarking on 30 binding-potency prediction tasks using activity-cliff datasets. Performance is measured by *R*^2^. Yellow stars indicate the top-performing model for each task.

Across the 30 activity-cliff tasks, CAMF achieved the best overall performance, reaching an average *R*^2^ of 0.665 (Tables 1 and S3). CAMF outperformed representative strong baselines, including MoLFormer, GROVER, Uni-Mol, and KPGT, by 7.5%, 11.8%, 8.7%, and 1.5%, respectively. Moreover, CAMF achieved the highest *R*^2^ on 16 of the 30 activity-cliff datasets and ranked second on 6 additional tasks (Fig. 2 and Table S3). These improvements were more pronounced than those observed on conventional property benchmarks, indicating that task-adaptive multimodal integration is especially beneficial when structural similarity is no longer a reliable surrogate for property similarity (Fig. 3).

**Fig. 3:**
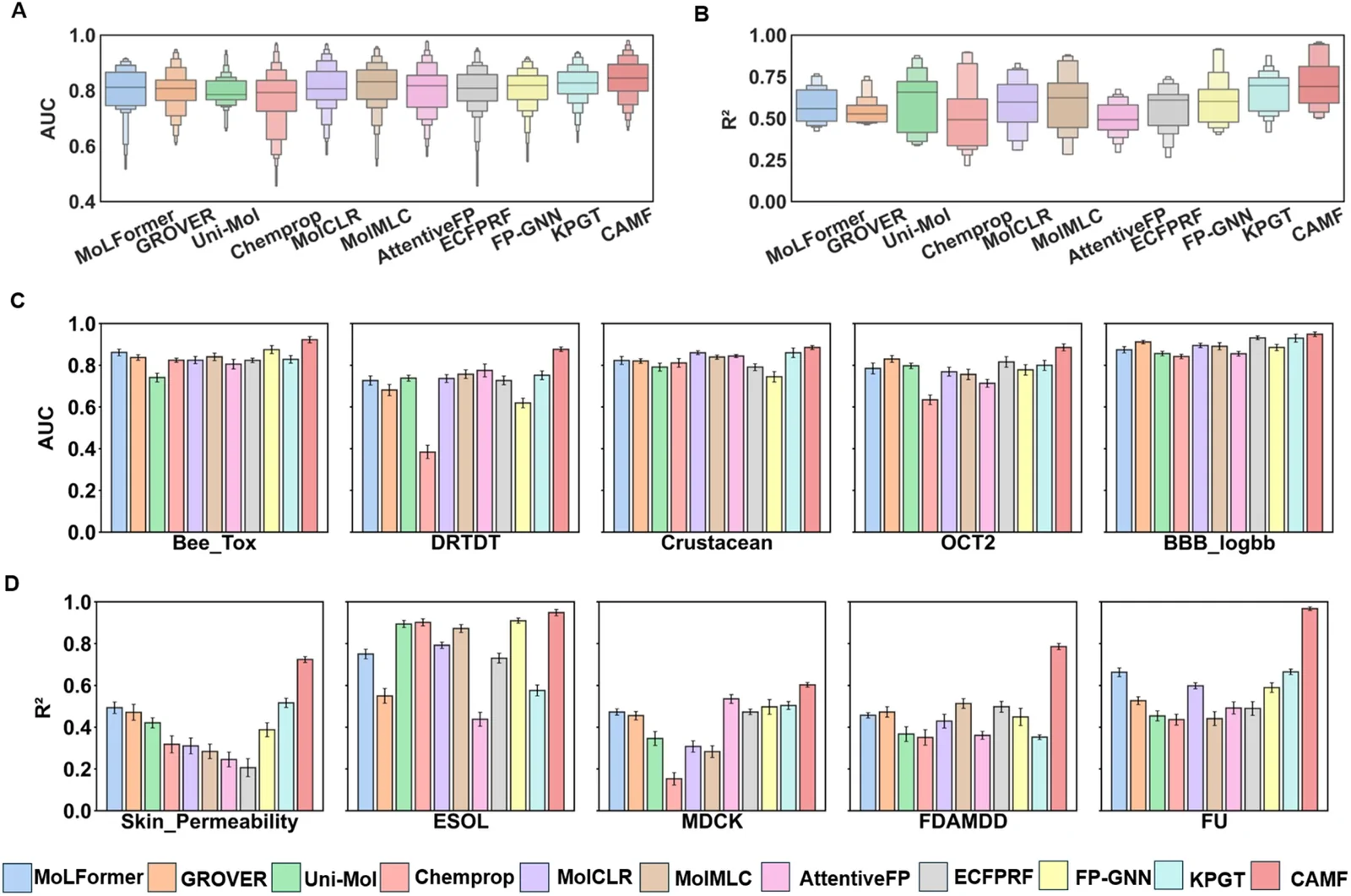
Evaluation of ADMET and physicochemical property datasets. (A) AUC across 62 classification tasks. (B) *R*^2^ across 15 regression tasks. (C) and (D) Performance in small-sample classification and regression settings, respectively.

To further dissect the source of this improvement, we compared the embedding-only variant (CAMF_emb) with the descriptor-based variant (CAMF_des). On the activity-cliff tasks, CAMF_des achieved a higher average *R*^2^ of 0.649 than CAMF_emb (0.586), suggesting that molecular descriptors provide particularly informative signals for modeling abrupt activity changes induced by subtle structural perturbations. A similar trend was observed for KPGT, which also incorporates descriptor features and ranked among the strongest baselines (Table S3). Together, these results indicate that accurate prediction on activity-cliff landscapes depends not only on learned embeddings, but also on integrating expert-defined structural descriptors that help recover property-relevant local differences. This observation further supports the central premise of CAMF: modality relevance is task-dependent, and non-smooth structure–activity relationships require selective integration of complementary molecular evidence rather than generic multimodal aggregation.

### 2.3. CAMF captures task-specific stereochemical requirements

Chirality-sensitive prediction provides a second stringent SSP setting because molecules with identical atomic composition and connectivity can exhibit distinct properties solely due to differences in stereochemical arrangement.^7^ To systematically evaluate stereochemical sensitivity, SSPBench includes two chirality-related tasks: (i) an *R*/*S* enantiomer classification benchmark comprising 31 270 single-chiral-center enantiomeric pairs (62 540 molecules) annotated according to the Cahn–Ingold–Prelog rules,^24^ and (ii) an optical rotatory strength regression benchmark built from 45 182 enantiomeric pairs, with labels computed using TD-DFT at the CAM-B3LYP/6-311++G(d,p) level. These two tasks probe distinct representation requirements: *R*/*S* assignment mainly depends on correct recognition of graph-defined stereocenters, whereas optical rotation is more strongly influenced by three-dimensional molecular geometry.^7,25^

Consistent with this distinction, models operating on 2D molecular graphs performed best on *R*/*S* classification. As shown in Table 2, representative graph-based models, including GROVER, MolCLR, and Chemprop, achieved near-perfect discrimination, approaching 100% ROC-AUC. In contrast, baselines that do not explicitly encode 2D graph stereochemistry, including RF (0D), MoLFormer (1D), and Uni-Mol (3D), performed close to random guessing (approximately 50% ROC-AUC), indicating limited ability to distinguish enantiomeric pairs from their native representations alone. Although CAMF is not itself a purely graph-based model, it achieved an ROC-AUC of 95.3%, showing that multimodal integration enables it to recover the graph-level stereochemical information required for chirality-sensitive classification.

**Table 2:** Performance comparison of baseline models and CAMF on chirality-sensitive prediction tasks

	Chirality *R*/*S* (AUC)	Rotation strength (MAE)
MoLFormer	0.728	0.0325
GROVER	0.993	0.0381
Uni-Mol	0.508	0.0324
Chemprop	**0.999**	0.0326
MolCLR	0.998	0.0332
MolMCL	0.998	0.0328
AttentiveFP	0.761	0.0376
RF	0.5	0.0403
FP-GNN	0.975	0.0374
KPGT	0.986	0.0317
CAMF	0.953	**0.0309**

A different pattern emerged for optical rotatory strength prediction, where 3D-aware representations became substantially more important. CAMF achieved the best overall performance and reduced MAE by 2.5–23.3% relative to the baselines, while Uni-Mol also outperformed most 2D graph models, consistent with the strong dependence of optical rotation on three-dimensional geometry. By incorporating 3D embeddings together with complementary 2D and descriptor-level information, CAMF remained effective across both chirality tasks and was particularly advantageous for stereochemistry-dependent regression. Together, these results show that chirality-sensitive prediction is strongly task-dependent: 2D graph representations are sufficient for explicit stereocenter assignment, whereas 3D geometry becomes critical for stereochemistry-dependent property prediction. This finding further supports the central premise of CAMF that accurate modeling of SSPs requires selective integration of complementary molecular evidence, rather than reliance on any single representation modality.

### 2.4. Conventional-property benchmarks reveal complementary modality preferences

Having established the advantages of CAMF in two stringent SSP settings, we next asked whether modality complementarity also benefits molecular property prediction in relatively smoother regimes. We therefore evaluated CAMF and ten baseline models on the conventional component of SSPBench, comprising 62 ADMET classification tasks and 15 physicochemical regression tasks. Among the unimodal baselines, MoLFormer, GROVER, and Uni-Mol emerged as the strongest encoders for the 1D, 2D, and 3D modalities, respectively, with mean classification AUCs of 80.2%, 80.0%, and 79.6%, and mean regression *R*^2^ values of 57.8%, 55.0%, and 59.0%. These results indicate that no single modality is uniformly optimal across conventional molecular property tasks and support the use of complementary encoders in CAMF.

Across the conventional benchmarks, CAMF achieved the best overall performance (Fig. 3). On the classification tasks, CAMF reached an average AUC of 84.1%, improving over the baselines by 1.8–7.3% and outperforming MoLFormer, GROVER, Uni-Mol, and KPGT by 3.9%, 4.1%, 4.5%, and 1.8%, respectively. On the regression tasks, CAMF attained an average *R*^2^ of 71.3%, representing gains of 5.8–21.4% over the baselines and improvements of 13.5%, 16.3%, 12.3%, and 5.8% relative to MoLFormer, GROVER, Uni-Mol, and KPGT, respectively (Fig. S2). Although these gains were smaller than those observed for the SSP tasks, they show that multimodal integration is beneficial not only under non-smooth structure–property relationships, but also in broader conventional-property prediction settings.

Both CAMF_emb and CAMF_des also delivered competitive performance, with CAMF_emb achieving an average AUC of 82.1% and *R*^2^ of 64.1%, and CAMF_des reaching 83.7% AUC and 67.9% *R*^2^ (Fig. 4). Together with the strong overall performance of CAMF, these results suggest that both learned embeddings and expert-defined descriptors provide useful and partly complementary signals for conventional molecular property prediction. Notably, the results on these conventional tasks already indicate that modality relevance is task-dependent, thereby providing a useful contrast to the more pronounced modality dependence observed in the SSP settings above. We next dissect how feature selection and adaptive fusion contribute to these performance gains.

**Fig. 4:**
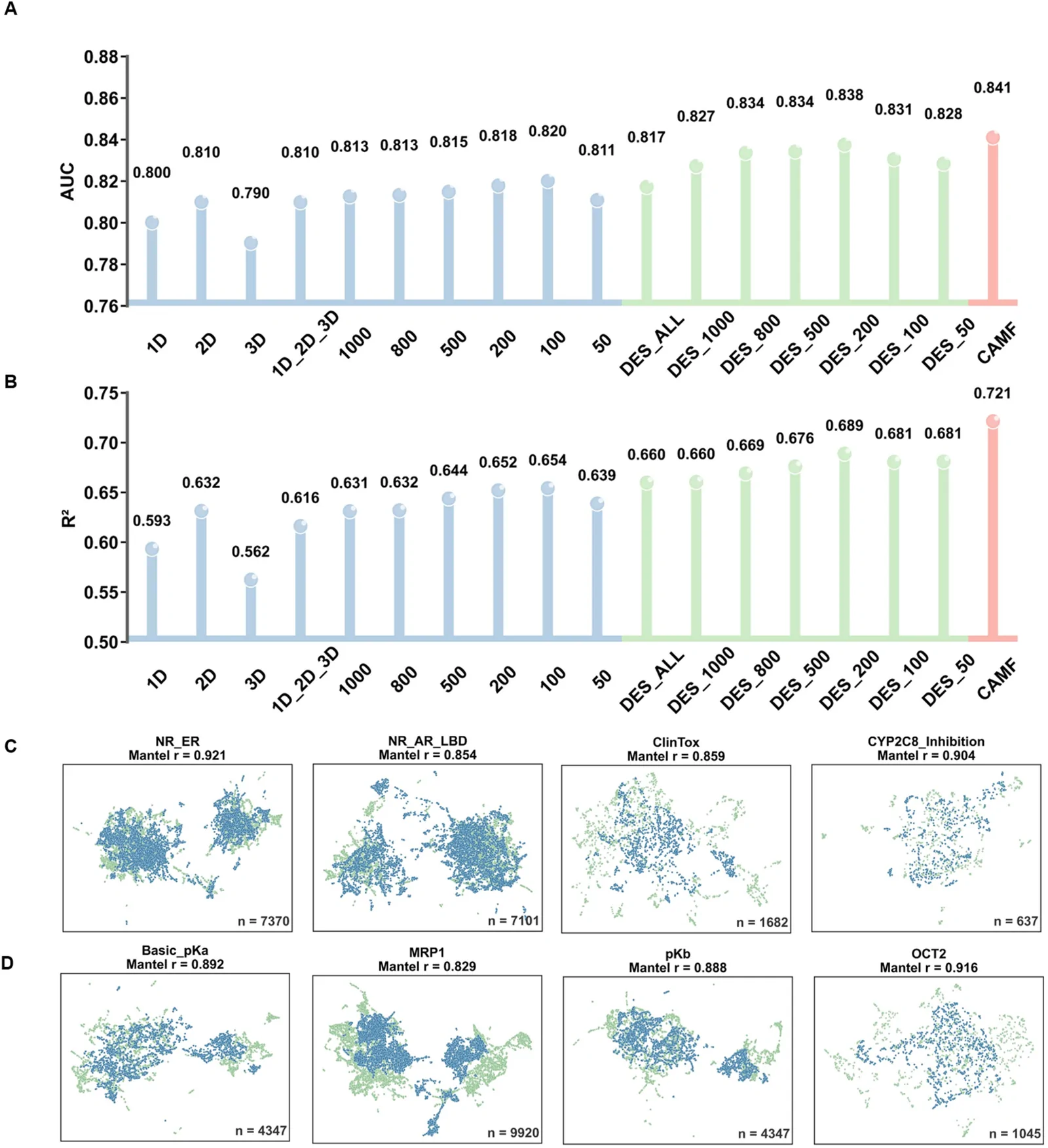
Impact of different input features on model performance. (A) AUC scores for classification tasks across models with varying feature sets. (B) *R*^2^ scores for regression tasks across models with varying feature sets. (Blue denotes the performance of CAMF_emb after FS; green denotes the performance of CAMF_des after FS.) (C) and (D) Present the UMAP projections and corresponding Mantel *r* values for 100-dimensional features and full-dimensional CAMF_emb features in classification and regression tasks, respectively.

### 2.5. Task-adaptive feature selection and fusion explain the gains of CAMF

To determine whether the gains of CAMF arise simply from larger multimodal representations or from selective integration of property-relevant molecular evidence, we performed ablation analyses at both the modality level and the framework level. Overall, these analyses show that CAMF benefits not from naive accumulation of features, but from task-adaptive prioritization of complementary structural signals across molecular modalities.

At the modality level, CAMF integrates embeddings from MoLFormer (1D), GROVER (2D), and Uni-Mol (3D) together with molecular descriptors, thereby combining sequence-level, graph-level, geometry-aware, and expert-defined structural information. This full modality-complementary representation consistently outperformed its reduced variants. Specifically, CAMF surpassed CAMF_emb by 2.0% in AUC and 7.1% in *R*^2^, and outperformed CAMF_des by 0.4% in AUC and 3.4% in *R*^2^ (Fig. 3 and 4). These results indicate that learned embeddings and molecular descriptors contribute partly distinct but complementary information. To further assess modality relevance, we restricted single-modality analyses to CAMF_emb in order to avoid confounding from multimodal descriptor information. Under this setting, 2D features achieved the strongest average performance on conventional-property tasks, reaching 81.0% AUC and 63.2% *R*^2^, and exceeding 1D features by 1.6% in AUC and 4.0% in *R*^2^, and 3D features by 1.7% in AUC and 7.2% in *R*^2^ (Fig. 4). Moreover, 2D features achieved the best performance on 40 of 62 classification tasks and 9 of 15 regression tasks (Fig. S3), with particularly notable gains for endpoints such as Cyp2B6_inhibitor, CYP2C8_Inhibition, CYP3A4_substrate, Basic_pKa, Caco2_reg, and LC50DM. These findings suggest that 2D graph information is often especially informative for conventional ADMET and physicochemical endpoints, while the best overall performance is only achieved when CAMF integrates complementary signals across all modalities. Together with the results from activity-cliff and chirality benchmarks, these analyses support the broader conclusion that modality relevance is inherently task-dependent.

At the framework level, the results further show that simple multimodal concatenation is insufficient and may even introduce feature redundancy. Without adaptive selection, multimodal features achieved classification performance comparable to the best unimodal setting, but regression performance remained weaker, indicating that merely increasing feature dimensionality does not guarantee better prediction (Fig. 4). To address this issue, CAMF incorporates random-forest-based feature selection (FS) as a core component. For CAMF_emb, concatenation of the three embedding spaces produced 1765 features, and reducing this representation to 100 selected features yielded the best average performance, improving AUC by 1.0% over the original multimodal representation and by 3.0% over unimodal inputs; for regression, the corresponding *R*^2^ improvements were 3.8% and 9.2%, respectively. For CAMF_des, the best performance was obtained with a 200-dimensional feature subset, reaching 83.8% AUC and 68.9% *R*^2^, which represented gains of 2.1% and 2.9% over the original descriptor-based multimodal representation (Fig. 4). Notably, these improvements were achieved using only approximately 10% of the original features, indicating that a substantial portion of the concatenated feature space is task-irrelevant or redundant. Consistent with this interpretation, UMAP projections and Mantel tests showed that the RF-selected feature subsets preserved the global structure of the original feature space, with Mantel *r* > 0.80 for tasks such as NR_ER, CYP2C8_Inhibition, and OCT2 (Fig. 4C and D). Thus, adaptive dimensionality selection improves predictive accuracy while retaining the core geometric organization of the multimodal representation.

We next evaluated whether the adaptive architecture of CAMF contributes beyond feature construction alone. Framework-level ablations showed that adding FS and attention-based fusion produced consistent improvements across conventional and structure-sensitive property tasks (Fig. 5A, C and E). Relative to unimodal baselines, the multimodal CAMF improved AUC by 5.1% and *R*^2^ by 15.9% on conventional-property tasks, and further increased *R*^2^ by 12.6% on SSP tasks. Adding domain-informed descriptors (CAMF_des) yielded an additional approximately 2.0% gain in AUC and 7.0–7.7% gain in *R*^2^. Importantly, when source-model embeddings were used within the CAMF framework, the resulting models consistently outperformed the corresponding original source models, with especially pronounced gains on regression tasks. For example, relative to the original GROVER model, CAMF-emb 2D w/FS improved regression performance by 5.9% on ADMET and physicochemical datasets and by 7.8% on activity-cliff tasks (Fig. 5B, D and F). These observations indicate that CAMF gains do not simply reflect stronger encoders, but arise from the combination of adaptive feature selection, reduced multimodal redundancy, and property-specific fusion of informative features. In detailed analyses of activity-cliff prediction, CAMF delivered markedly larger improvements than single-modality features without the adaptive framework, with several tasks showing gains of at least 10 percentage points in *R*^2^ and some exceeding 20 percentage points (Fig. 5G). Collectively, these results provide systematic evidence that 1D, 2D, 3D, and descriptor-based molecular representations are complementary across property regimes, and that CAMF's task-adaptive framework is essential for converting this complementarity into robust gains, particularly in challenging SSP settings.

**Fig. 5:**
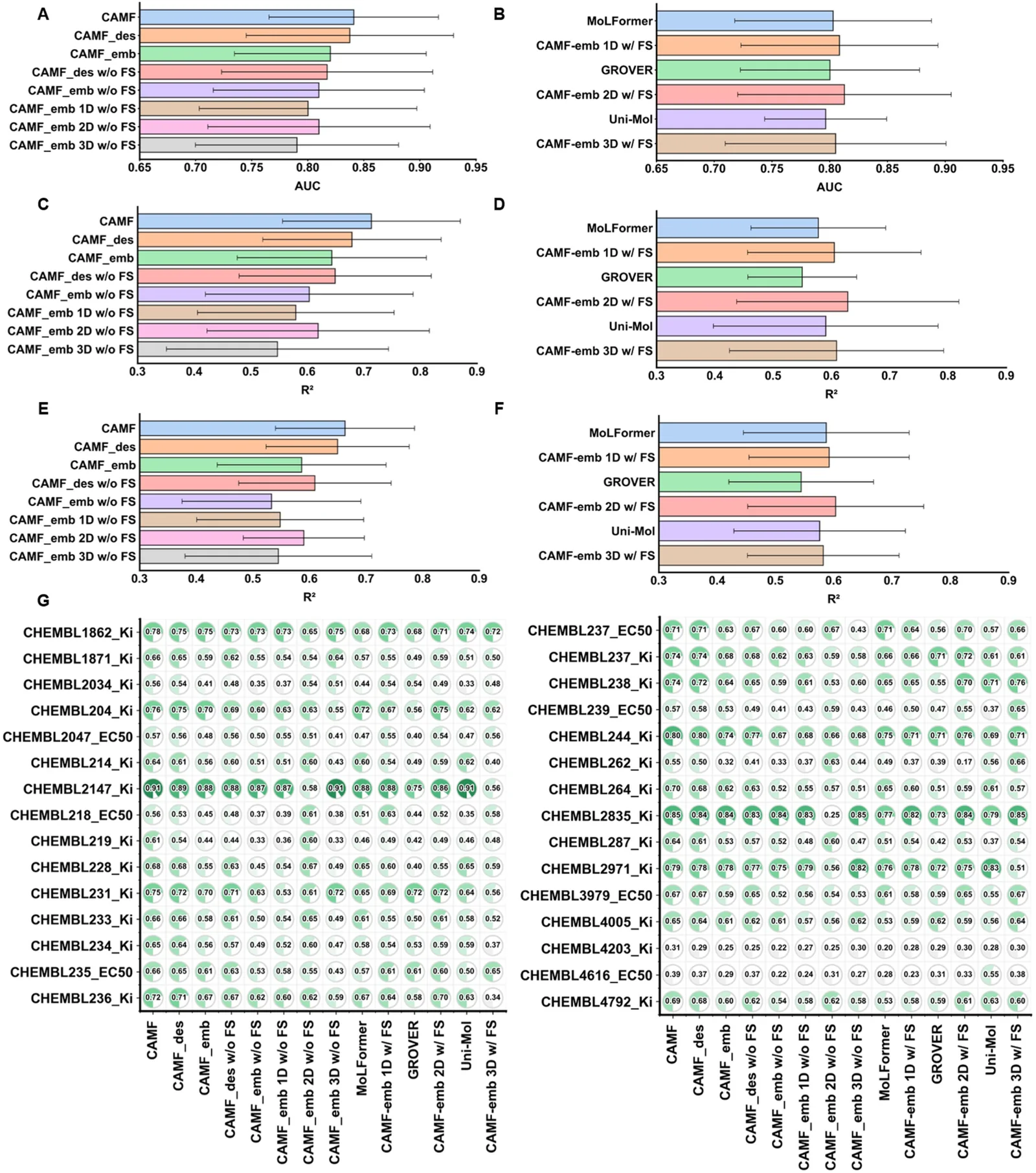
Ablation study of the CAMF framework. (A), (C) and (E) Performance of the full CAMF model and its ablated variants on classification tasks from the ADMET and physicochemical datasets, regression tasks from the ADMET and physicochemical datasets, and activity-cliff tasks, respectively. (B), (D) and (F) Performance comparisons between representative unimodal baselines and their CAMF-enhanced variants on classification tasks from the ADMET and physicochemical datasets, regression tasks from the ADMET and physicochemical datasets, and activity-cliff tasks, respectively. (G) Task-wise *R*^2^ on activity-cliff datasets. “w/o FS” denotes models without the FS module, and “w/FS” denotes models with the FS module.

### 2.6. Mechanistic interpretability of CAMF in activity-cliff prediction

To further probe CAMF's sensitivity to activity shifts induced by subtle structural perturbations, we applied SHAP analysis to the descriptor-based variant (CAMF_des) and examined representative docking poses generated with Schrödinger Glide 2024.^26^ We selected three thrombin-targeting activity-cliff compounds—CHEMBL366556, CHEMBL369583, and CHEMBL177142—with reported activities of 4610 nM, 2670 nM, and 32 nM, respectively, against thrombin (PDB ID: 2BXT).^27^ CAMF predicted their activities with accuracies of 83.6%, 88.1%, and 90.1%, respectively (Fig. 6A), indicating that it remained sensitive to pronounced activity changes arising from subtle structural differences.

**Fig. 6:**
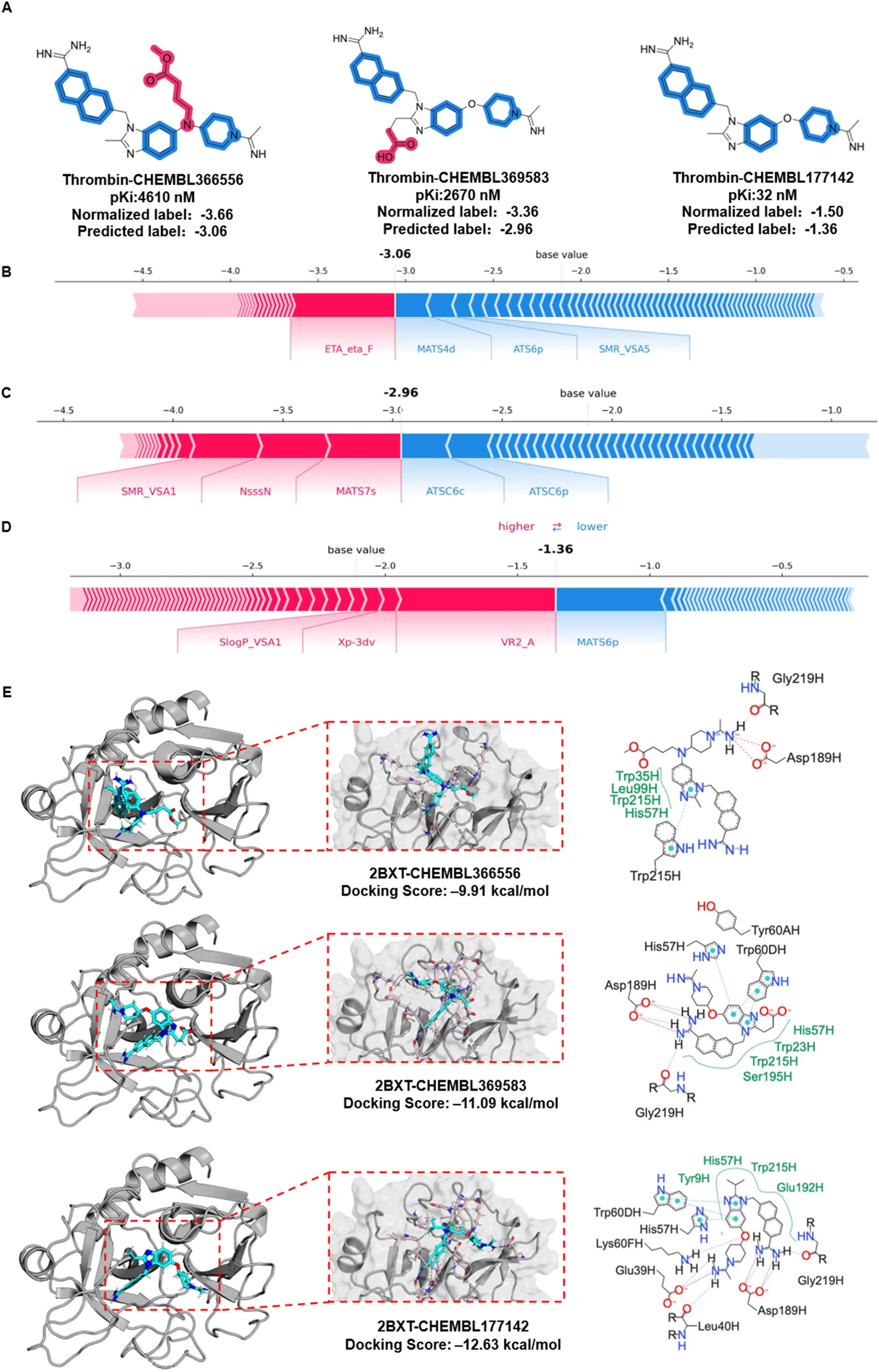
CAMF interpretability for three thrombin-targeting compounds using SHAP analysis and docking poses (CHEMBL366556, CHEMBL369583, and CHEMBL177142). (A) Names, experimental activities, normalized labels, and predicted labels. (B)–(D) Feature contribution analysis for three active compounds using SHAP. Positive contributions are shown in blue and negative contributions are shown in red. (E) Binding poses of the three active compounds targeting thrombin.

SHAP analysis identified several descriptors with lag = 6, including ATSC6C, ATSC6p, ATS6p, and MATS6p, as major contributors to model predictions (Fig. 6B–D). These descriptors typically reflect atom-pair relationships separated by six bonds and are often associated with aromatic six-membered rings.^28^ In the three active compounds, these descriptors were positively associated with activity, suggesting that aromatic ring motifs are important determinants of thrombin-targeting efficacy. Consistent with this interpretation, docking analysis showed that these aromatic fragments form favorable π–π stacking and hydrophobic interactions with residues in the thrombin binding pocket, thereby stabilizing ligand binding. This observation is also in line with previous reports highlighting the importance of aromatic scaffolds for thrombin inhibition.^29^

In addition, the descriptor NssN suggested that nitrogen-containing side chains may reduce activity (Fig. 6C). Docking analysis of CHEMBL369583 and CHEMBL366556 indicated that introducing a nitrogen substituent perturbed the orientation of the benzimidazole ring, weakened π–π stacking interactions, and decreased the predicted binding affinity from −11.09 to −9.91 kcal mol^−1^. Likewise, the hydrophilic surface-area descriptor SlogP_VSA1 showed a negative association with predicted activity (Fig. 6D). CHEMBL366556 contains a hydrophilic side chain, leading to a higher SlogP_VSA1 value and reduced binding affinity, whereas CHEMBL177142 lacks this moiety and displayed substantially stronger activity. Docking against thrombin further supported this trend, with the predicted binding affinity improving from −11.09 kcal mol^−1^ for CHEMBL369583 to −12.63 kcal mol^−1^ for CHEMBL177142 (Fig. 6E). Given that the thrombin pocket is enriched in hydrophobic residues such as Trp215, removal of polar substituents likely strengthens hydrophobic packing and reduces steric interference, thereby enhancing activity. Together, these results indicate that CAMF not only predicts activity-cliff behavior accurately but also recovers chemically meaningful substructures and interpretable structure–activity signals underlying local activity shifts.

### 2.7. Retrospective case studies on clinically reported safety liabilities

To provide a retrospective assessment of whether CAMF predictions are concordant with clinically reported safety liabilities, we analyzed five compounds, including investigational agents and previously approved drugs that were ultimately associated with clinically significant liabilities across multiple ADMET endpoints. These compounds were absent from the training data and showed Tanimoto similarities below 0.6 to molecules in the corresponding benchmark datasets. These compounds included tegaserod, a 5-HT4 receptor agonist withdrawn because of cardiovascular risk; rofecoxib, a selective COX-2 inhibitor withdrawn because of increased risks of myocardial infarction and stroke; regorafenib, an oral multi-kinase inhibitor carrying a boxed warning for severe hepatotoxicity; fialuridine (FIAU), whose clinical development was halted after fatal liver failure; and obeticholic acid (OCA), which has been associated with severe liver injury (Table 3).^30–34^

**Table 3:** Retrospective comparison of cardiotoxicity-related predictions for rofecoxib and tegaserod^a^

Drug	Tool	Cardiotoxicity-10	Cardiotoxicity-30
Rofecoxib	CAMF	Alert	Alert
	admetSAR 3.0	Non-alert	Non-alert
	ADMETlab 3.0	Alert	N/A
Tegaserod	CAMF	Alert	Alert
	admetSAR 3.0	Alert	Alert
	ADMETlab 3.0	Alert	N/A

a “Alert” indicates a predicted toxicity alert; “non-alert” indicates no predicted alert; “N/A” indicates that the corresponding endpoint is not provided by the tool.

We compared CAMF with two widely used ADMET prediction tools, ADMETlab 3.0 and admetSAR 3.0, for cardiotoxicity- and hepatotoxicity-related predictions for these compounds.^35,36^ CAMF predictions were concordant with the reported safety liabilities in these selected retrospective examples. CAMF flagged both rofecoxib and tegaserod for the Cardiotoxicity-10 and Cardiotoxicity-30 endpoints. For hepatotoxicity, CAMF flagged regorafenib, fialuridine, and obeticholic acid for hepatotoxicity-related endpoints. Although all three tools flagged regorafenib and fialuridine for hepatotoxicity-related endpoints, obeticholic acid was not flagged by ADMETlab 3.0 (Table S4).

Overall, these retrospective case studies highlight the potential of CAMF to support safety-risk prioritization in drug discovery. By combining robust molecular representations with task-adaptive feature selection and multimodal fusion, CAMF flagged clinically reported cardiotoxicity- and hepatotoxicity-related liabilities in selected representative compounds. While these analyses should not be interpreted as independent prospective validation, they illustrate how CAMF can help prioritize compounds warranting closer safety evaluation during early-stage drug discovery.

## 3. Conclusion

Structure-sensitive properties expose a fundamental challenge in molecular machine learning because small structural perturbations can induce disproportionately large changes in molecular behavior, thereby breaking the smooth structure–property assumptions that underlie many existing representation-learning strategies. In this work, we show that accurate modeling of SSPs depends not on any single molecular representation, but on the task-adaptive integration of complementary structural evidence. Across activity-cliff and chirality-sensitive benchmarks, as well as conventional molecular property tasks, our results consistently indicate that modality relevance is strongly task-dependent: 2D graph information is often highly informative for conventional ADMET and stereocenter assignment, whereas descriptors and 3D geometry become especially important for activity-cliff and stereochemistry-dependent property prediction.

To systematically examine this problem, we constructed SSPBench, a benchmark spanning both conventional molecular property prediction tasks and dedicated SSP settings, and developed CAMF, a task-adaptive modality-complementary framework that integrates MoLFormer, GROVER, Uni-Mol, and Mordred descriptors through feature selection and adaptive fusion. Rather than relying on naive multimodal concatenation, CAMF selectively prioritizes property-relevant molecular evidence while suppressing redundant or task-irrelevant signals. This design enables CAMF to achieve robust and accurate performance across diverse property regimes, with particularly strong gains in challenging SSP tasks. Mechanistic analyses further show that CAMF can recover chemically meaningful determinants of activity changes and identify clinically relevant toxicity liabilities in representative case studies, supporting both its interpretability and practical utility.

CAMF nevertheless has important limitations. First, multimodal feature generation remains computationally expensive, especially when multiple pretrained encoders and descriptor calculations are combined. Second, although feature attribution and descriptor analysis improve interpretability, precise localization of activity-determining substructures remains indirect and could be strengthened by future atom- or fragment-level explanation strategies. Despite these limitations, our study provides a broader conceptual message: non-smooth structure–property relationships require selective integration of complementary molecular modalities, rather than reliance on a single representation family or generic multimodal aggregation. We expect that this principle will be useful not only for molecular property prediction, but also for future multimodal learning problems involving more complex molecular and biomolecular systems.

## 4. Methods

### 4.1. Benchmark datasets for structure-sensitive molecular property prediction

SSPBench was constructed to evaluate molecular property prediction under structure-sensitive settings, with emphasis on whether models can capture subtle structure–property relationships. It integrates routine ADMET and physicochemical tasks with activity-cliff and chirality-sensitive benchmarks. The ADMET and physicochemical subset was compiled from peer-reviewed studies and public ADMET resources and contains 77 tasks (62 classification and 15 regression), covering endpoints such as solubility, permeability, metabolic stability, half-life, and toxicity, with more than 197 438 molecules in total.^35–39^ To explicitly probe structural sensitivity, SSPBench further includes 30 activity-cliff tasks spanning 48 707 molecules across diverse target families, including kinases, nuclear receptors, G protein-coupled receptors, transferases, and proteases.^8^ In addition, chirality-sensitive benchmarks were constructed from 131 000 chiral molecules collected from PubChem, yielding 45 182 enantiomeric pairs. Among them, 31 270 single-chiral-center pairs (62 540 molecules) were annotated as *R* or *S* for binary classification, while the full set of 45 182 pairs was used for regression of TDDFT-computed optical rotatory strengths.

Scaffold splits were used for the ADMET, physicochemical, and activity-cliff tasks to maximize structural dissimilarity between training and test sets, whereas chirality-related tasks used enantiomer-stratified random splits. All models were evaluated under a unified protocol.

### 4.2. Feature construction module

#### 4.2.1. 1D feature extraction

MoLFormer^21^ comprises 12 transformer encoder layers, where each layer contains 12 attention heads and the hidden size is set to 768. Self-attention enables the network to construct complex representations that incorporate context from the whole molecular sequence. Attention mechanisms transform the sequence features into query (*Q*), keys (*K*) and value (*V*) representations, as shown below:1
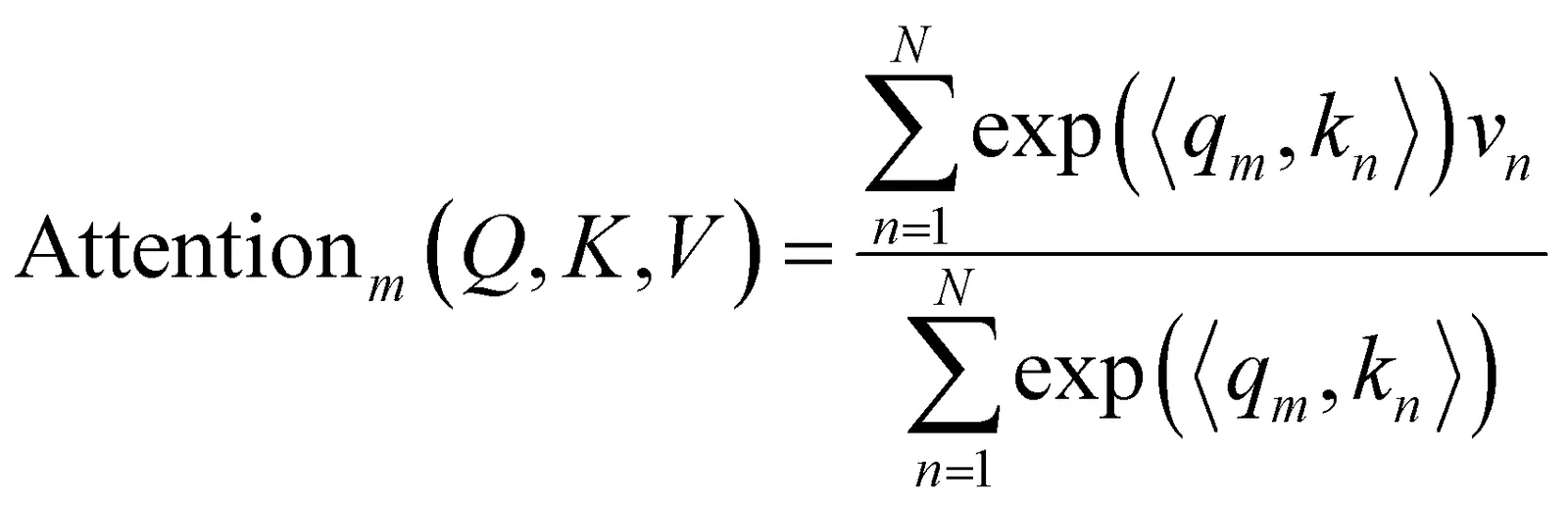


#### 4.2.2. 2D feature extraction

GROVER^20^ is a self-supervised graph transformer pretrained on a large-scale molecular dataset. We employed GROVER-large to obtain a graph representation with a dimensionality of 485. A molecule is represented as *G*(*V*, *E*), where *V* is the atom set and *E* i is the bond set. Each atom *i* ∈ *V* is initialized with a feature vector *h*^(0)^_*i*_ containing atomic descriptors, while each bond (*i*, *j*) ∈ *V* is initialized with an edge feature *e*^(0)^_*ij*_. In each message-passing layer *l*, atom features are updated as:2
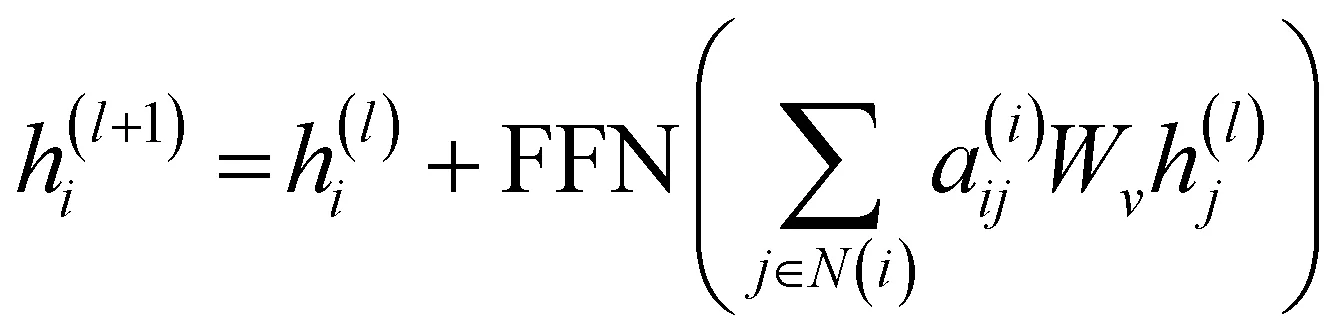
where *h*^(*l*)^_*i*_ is the atom feature at layer *l*, *N*(*i*) is the neighbor set of atom *i*, *W*_*v*_ is the value projection matrix and *a*^(*i*)^_*ij*_ is the attention coefficient between atoms *i* and *j*, computed by:3
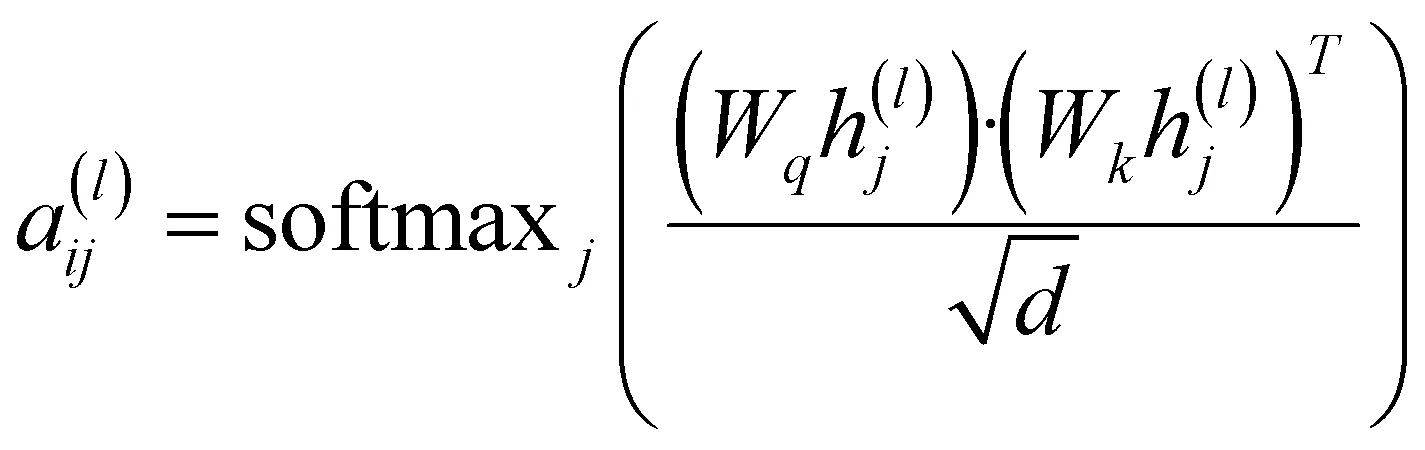
where *W*_*q*_ and *W*_*k*_ are the query and key projection matrices and *d* is the hidden dimension. After *l* layers, node representations are aggregated *via* an attention-based readout to obtain the graph-level embedding *h*_G_.

#### 4.2.3. 3D feature extraction

Uni-Mol^22^ employs rotation and translation-invariant spatial encoding with atom-pair representations to model the 3D molecular structure. We extract a 512-dimensional 3D embedding and incorporate it into CAMF. To encode continuous coordinates, Uni-Mol computes pairwise Euclidean distances and expands them with Gaussian radial bases to obtain SE(3)-invariant spatial features. The model maintains a pair-level representation in each layer, which is updated to use multi-head self-attention *via* the query–key dot product as follows:4
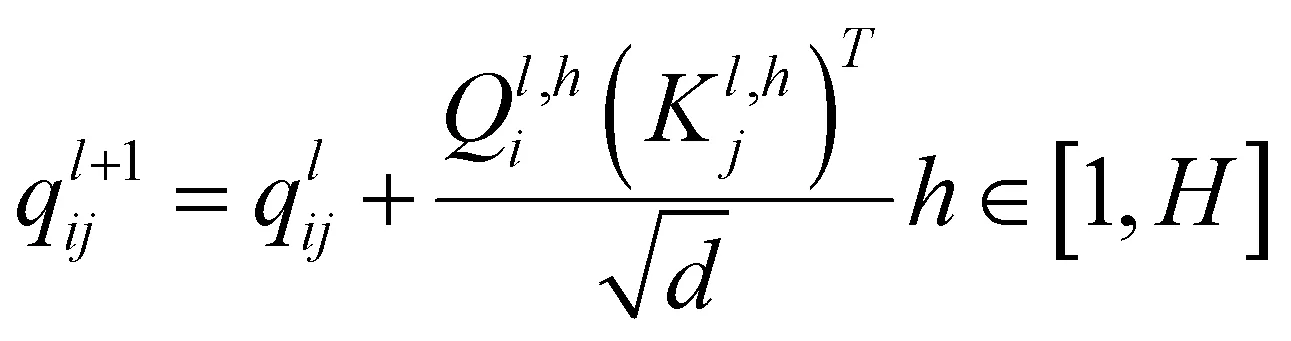
where *q*^*l*+1^_*ij*_ is the pair representation of atom pair *ij* in the *ij*-th layer; *H* is the number of attention heads; *d* is the dimension of hidden representations; *Q*^*l*,*h*^_*i*_and *K*^*l*,*h*^_*i*_are the query and key of the *i*-th (*j*-th) atom in the *l*-th layer and *h*-th head.

#### 4.2.4. Molecular descriptor extraction

Molecular descriptors are computed using Mordred.^28^ It converts molecular structures into numerical features by computing over 1800 standardized descriptors spanning physicochemical, structural, and topological properties, such as, ABC (atom-bond connectivity index), SpAbs_A (SpAbs of the adjacency matrix), and SpAD_A (SpAD of the adjacency matrix). These interpretable, domain-informed descriptors complement learned embeddings in CAMF and are used as the descriptor modality (CAMF_des) in Fig. 1B.

### 4.3. Adaptive feature selection

In this study, CAMF uses RF models to evaluate the importance of features by assessing each feature's contribution to variance reduction.^23^ Specifically, feature importance is quantified based on the reduction in node impurity, measured by the Gini impurity index, at each split of the RF.^40^ Each time a feature is chosen to split the data points during training, the reduction in impurity is added to the importance score of that feature. The overall feature importance is computed as follows:5
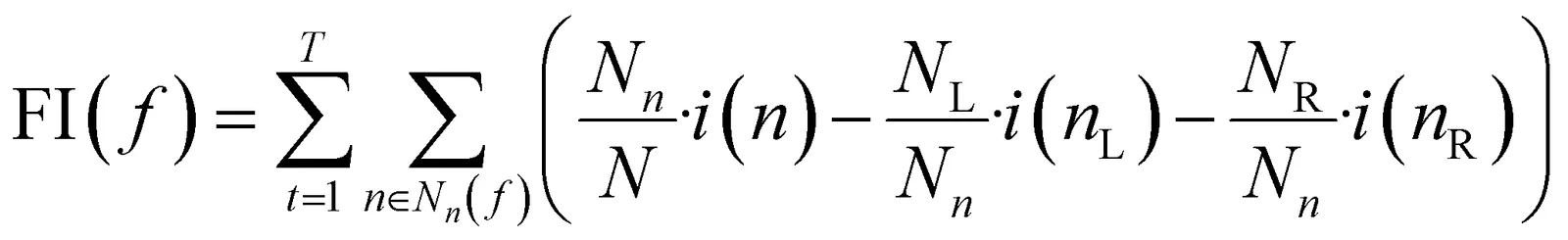
where FI(*f*) is the feature importance score for feature *f*; *T* is the number of trees in the forest; *n* ∈ *N*_*n*_(*f*) is the set of nodes in the tree where feature *f* is used for splitting; *N* is the total number of data points; *i*(*n*) is the impurity at node *n*; *N*_L_ and *N*_R_ are the number of data points in the left and right child nodes; *i*(*n*_L_) and *i*(*n*_R_) are the impurity values of the left and right child nodes.

### 4.4. Evaluation metrics

For classification tasks, performance was assessed using the area under the receiver operating characteristic curve (AUC). For regression tasks, we report the coefficient of determination (*R*^2^) and the mean absolute error (MAE). Higher values are better for AUC and *R*^2^, whereas lower values indicate better performance for MAE.

### 4.5. Baselines

We evaluated CAMF against ten baselines spanning 1D, 2D, 3D, and multimodal representations. (1) Chemprop,^16^ a supervised directed message-passing neural network (d-MPNN) that learns graph embeddings from 2D molecular graphs for MPP. (2) AttentiveFP,^17^ a supervised graph attention network with gated message passing and attentive readout on 2D molecular graphs. (3) FP-GNN^18^ is a fingerprint-guided GNN that fuses fingerprints with graph features to steer message passing. (4) MolCLR^19^ employs contrastive pretraining on molecular graphs using augmentations (*e.g.*, atom/bond perturbation and subgraph masking). (5) MolMCL^9^ is a multi-level contrastive pretraining framework for molecular graphs that unifies attribute-masking objectives with parameter-perturbed view generation to learn transferable chemical representations. (6) GROVER^20^ is a self-supervised graph transformer pretrained on large-scale molecular graphs, yielding chemistry-informed 2D molecular-graph representations. (7) MoLFormer,^21^ is a transformer pretrained with a masked language modeling (MLM) objective, yielding SMILES-based molecular embeddings. (8) Uni-Mol^22^ is an SE(3)-equivariant, 3D-aware transformer that leverages molecular conformers and is pretrained on coordinate recovery and masked-atom prediction to capture spatial structure. (9) KPGT^14^ is a knowledge-guided graph transformer that injects descriptors and fingerprints *via* a knowledge node during pretraining. (10) ECFP_RF^41^ is a classical baseline that couples extended-connectivity fingerprints (ECFPs) with a RF. We re-evaluated all models on the same splits. All other settings—including model hyperparameters and training procedures—were kept at the defaults of the respective baselines to ensure a fair and reproducible comparison.

## Author contributions

Conceptualization: X. Y., C. G., and S. L. Methodology: S. L., S. Z., and S. W. Software: S. L., S. Z., and S. W. Investigation: S. L., S. Z., S. W., X. Z., W. L., L. Q., L. S., K. X., and K. Z. Data curation: L. S., K. X., and K. Z. Visualization: X. Z., W. L., and L. Q. Funding acquisition: H. L. and X. Y. Project administration: H. L. and X. Y. Supervision: X. Y., C. G., and H. L. Writing – original draft: S. L., S. Z., and S. W. Writing – review & editing: Y. L., Y. T., X. Y., C. G., H. L., and all authors.

## Conflicts of interest

The authors declare that they have no known competing financial interests or personal relationships that could have appeared to influence the work reported in this paper.

## Supplementary Material

SC-017-D6SC02982E-s001

## Data Availability

The SSPBench datasets used in this study are available at https://github.com/linshaolong11/CAMF and https://zenodo.org/records/21253187. The bioactivity dataset was obtained from MoleculeACE (https://github.com/molML/MoleculeACE). Source data underlying the figures and tables in this paper are provided with the article. Code availability: the necessary data, code for reproducing the results, as well as documentation and usage examples, are available on GitHub at https://github.com/linshaolong11/CAMF. Supplementary information (SI): detailed benchmark dataset and task descriptions, data distributions and evaluation metrics, supplementary benchmark results, and case studies. See DOI: https://doi.org/10.1039/d6sc02982e.
