# Supplementary material for: Task-adaptive multimodal molecular representations for structure-sensitive property prediction
Source: Chem Sci. 2026 Sep 2;17(41):19964–77. doi: 10.1039/d6sc02982e (PMC13551429; doi:10.1039/d6sc02982e)
Supplement: Supplementary file 1 [file SC-017-D6SC02982E-s001.pdf]

Supplementary Information (SI) for Chemical Science.
This journal is © The Royal Society of Chemistry 2026

Supplementary Information

Task-adaptive multimodal molecular representations for structure-sensitive property prediction

Content

Supplementary Information I
Task-adaptive multimodal molecular representations for structure-sensitive property prediction I
Content II
1. Summary of ADMET and physicochemical property 1
Table S1. Summary of ADMET and physicochemical property endpoints, including task name, brief description, dataset size, task type, and references 1
2. The data distribution and scoring metrics for all bioactivity tasks 13
Table S2. Data distribution and scoring metrics for all bioactivity tasks 13
Figure S1. Task distribution of the large-scale molecular property dataset across eight categories: Absorption, Distribution, Metabolism, Excretion, Toxicity, Physicochemical, Bioactivity, and Chirality. 15
3. The performance of CAMF comparing baseline models 16
Figure S2. Heatmaps comparing model performance. Panel a shows AUC across multiple classification tasks. Panel b shows R2 across multiple regression tasks. 16
Figure S3. Heatmaps comparing model performance. a shows AUC classification tasks. b shows R^2 regression tasks. 17
5. The performance of CAMF comparing baseline in activity cliff **18**
Table S3. Performance (R^2) of 10 methods on 30 binding potency prediction tasks in activity cliff. 18
6. Mechanistic and translational case studies support practical utility 19
Table S4. The evaluation of Regorafenib, Fialuridine, and Obeticholic across three tools of cardiotoxicity of Hepatotoxicity 19
References **20**

1. Summary of ADMET and physicochemical property

Table S1. Summary of ADMET and physicochemical property endpoints, including task name, brief description, dataset size, task type, and references

Task	Task explanation	Dataset Size	Task Type	ref
	**Absorption**			
Bioavailability	Predicting whether a drug can achieve adequate oral bioavailability in humans (i.e., reach systemic circulation after oral dosing).	632	Classification	[1,2]
F20%	Predicting whether a drug achieves ≥ 20% human oral bioavailability.	993	Classification	[3]
F30%	Predicting whether a drug achieves ≥ 30% human oral bioavailability.	980	Classification	[3]
F50%	Predicting whether a drug achieves ≥ 50% human oral bioavailability.	1,447	Classification	[3]
OB	Estimating the fraction of an orally administered dose that reaches systemic circulation (oral bioavailability), reported as a percentage.	994	Classification	[4]
Pgp_inhibitor	Predicting whether a compound inhibits P-glycoprotein (P-gp; MDR1/ABCB1), an ATP-binding cassette efflux transporter impacting drug disposition.	1,948	Classification	[5]
Pgp_substrate	Predicting whether a compound is a substrate of P-glycoprotein (P-gp; MDR1/ABCB1), indicating it is actively transported by this efflux pump.	1,207	Classification	[6]
MDCK	Madin−Darby Canine Kidney cells (MDCK) have been developed as an in vitro model for permeability screening. Its apparent permeability coefficient, Papp, is widely considered to be the in vitro gold standard for assessing the uptake efficiency of chemicals into the body.	1,136	Regression	[7]
Caco2_reg	Caco-2 is a human colon adenocarcinoma cell line that differentiates into an enterocyte-like monolayer and is used as an in vitro intestinal barrier model. Caco-2 is a key proxy for oral absorption and can also reveal transporter effects.	2,946	Regression	[8]

**Distribution**				
BBB_logbb	The permeability of drugs through the blood-brain barrier (BBB) represents an important property in designing drugs that targets the central nervous system	1,216	Classification	[9]
BBBP	BBBP :Predict whether a compound is BBB permeant (class 1) or non-permeant (class 0). BBB-positive compounds are desirable for CNS targets; BBB-negative compounds are preferred for peripheral targets to limit CNS adverse effects	2,329	Classification	[10]
BCRP	BCRP (Breast Cancer Resistance Protein) inhibition: Predict whether a compound inhibits the efflux transporter BCRP (ABCG2), which mediates multidrug resistance and drug-drug interactions. BCRP inhibition can alter exposure of co-medications and impact safety and efficacy.	830	Classification	[11]
Oatp1b1	OATP1B1 inhibition: Predict inhibition of the hepatic uptake transporter OATP1B1 (SLCO1B1), a key mediator of liver drug clearance. OATP1B1 inhibition can cause clinically relevant drug-drug interactions and elevated systemic exposure	1,606	Classification	[12]
Oatp1b3	OATP1B3 inhibition: Predict the fraction of drug unbound to plasma proteins. Only unbound drug is free to distribute, reach targets, and be cleared; fu influences exposure, efficacy, and interaction risk.	1,501	Classification	[13]
Fu	Fu(Fraction unbound in plasma): Predict the fraction of drug unbound to plasma proteins. Only unbound drug is free to distribute, reach targets, and be cleared; fu influences exposure, efficacy, and interaction risk.	1,948	Regression	[14]
MRP1	MRP1 (Multidrug Resistance Protein 1) inhibition: Predict inhibition of the efflux transporter MRP1 (ABCC1), which affects distribution across tissues and barriers. MRP1 inhibition can modify pharmacokinetics of many substrates and contribute to drug-drug interactions.	1,207	Regression	[15]
PPB	PPB (plasma protein binding): Predict the extent of plasma protein binding of a compound. Higher binding reduces the unbound fraction	3,146	Regression	[16]

OCT2	available for pharmacologic action and clearance, impacting half-life, exposure, and interaction potential.<br>In mammals, organic cation transporter (OCT) subtypes 1 and 2 (OCT1 and OCT2, also known as SLC22A1 and SLC22A2, respectively) are poly specific transporters responsible for the uptake and clearance of structurally diverse cationic compounds in the liver and kidneys, respectively.	1,045	Classification	17

**Metabolism**				
CYP1A2_substrate	Drug biotransformation is commonly divided into two stages: phase I (functionalization) and phase II (conjugation). Phase I reactions are largely catalyzed by cytochrome P450 (CYP) enzymes. Humans encode 57 CYP isozymes, with a subset—CYP1A2, CYP3A4, CYP2C9, CYP2C19, CYP2D6, CYP2B6, and CYP2C8—responsible for most drug metabolism. These enzymes are predominantly expressed in the liver. While CYP-mediated metabolism facilitates drug elimination, it can also generate toxic or reactive intermediates and precipitate clinically relevant drug–drug interactions via inhibition, induction, or substrate competition.	439	Classification	18
CYP2B6_inhibitor		618		19
CYP2B6_substrate		2,892		20
CYP2C19_substrate		316		21
CYP2C8_inhibition		637		22
CYP2C9_substrate		765		23
CYP2D6_substrate		718		24
CYP3A4_substrate		1,415		25
HLM_Stability	The human liver microsome (HLM) stability assay is a widely used in-vitro method to assess hepatic metabolic stability and clearance. Using pooled HLMs (typically with NADPH), the assay tracks the loss of parent compound and/or formation of metabolites over time, yielding intrinsic clearance and half-life estimates that inform prediction of human hepatic clearance.	6,927	Classification	26
UGT_substrate	UGTs (UDP-glucuronosyltransferases) are phase II metabolic enzymes that catalyze	1,469	Classification	27

	drug glucuronidation mainly in the liver and intestine. A UGTs substrate is a compound that can be glucuronidated by these enzymes (common isoforms include UGT1A1, UGT1A9, UGT2B7).			

	**Excretion**			
$T_{1/2}$	The elimination half-life (t1/2) is the time required for the plasma drug concentration or the total amount of drug in the body to decrease by 50%. The t1/2 determines the dosing interval, the time to reach steady state and to complete washout (approximately 4-5 half-lives), the extent of accumulation, the washout period, and the risk of drug-drug interactions; it is therefore critical for designing dosing regimens and long- or short-acting formulations.	800	Classification	[28]

	**Toxicity**			
Ames	The Ames test is an in vitro mutagenicity screening assay that employs Salmonella typhimurium strains carrying defined gene mutations to detect compound-induced reverse mutations, with or without metabolic activation, thereby indicating potential genotoxicity.	7,177	Classification	[29]
Avian_Tox	Refers to the acute or chronic toxicity of a chemical to birds (commonly modeled by mallard duck and quail). High avian toxicity indicates substantial ecological risk and non-target organism harm, influences overall chemical safety assessment and life-cycle exposure evaluation, and may signal cross-species toxicity mechanisms.	697	Classification	[30]
Bee_Tox	Refers to toxicity toward pollinators, primarily the honeybee Apis mellifera. High bee toxicity indicates harm to the critical ecological function of pollination and affects the overall safety profile of a chemical and acceptable environmental exposure levels.	218	Classification	[31]
Bace	BACE1 (beta-site APP cleaving enzyme 1) is an	1,750	Classification	[32]

	aspartyl protease that mediates beta-cleavage of APP and is a key target for amyloid-beta production in Alzheimer's disease. Task: predict whether a compound is a BACE1 inhibitor (binary classification: active vs inactive), commonly used in early CNS drug screening.			
Biodegradation	The capacity and rate at which a chemical is broken down in the environment by biological processes. High biodegradability generally implies lower environmental persistence and reduced risks of long-term exposure and bioaccumulation.	1,860	Classification	[33]
Carcinogenicity	The potential of a chemical to induce tumors, encompassing both genotoxic (direct DNA damage) and non-genotoxic mechanisms. Carcinogenicity directly informs long-term human safety risk, risk stratification, and regulatory classification.	1,716	Classification	[34]
Cardiotoxicity-1<br>Cardiotoxicity-5<br>Cardiotoxicity-10<br>Cardiotoxicity-30	Cardiotoxicity-1/5/10/30 are four binary endpoints that predict whether a compound is cardiotoxic at in-vitro assay concentrations of 1, 5, 10, and 30 uM (e.g., cardiomyocyte or ion-channel assays). Together they characterize the dose–response profile: positivity at lower concentrations indicates greater intrinsic cardiotoxic liability, whereas a shift from negative to positive only at higher concentrations suggests risk under high exposure or drug–drug interaction–mediated exposure increases.	1,702	Classification	[35,36]
Clinical_Toxicity	Flags compounds linked to clinical toxicity in humans, reflecting real-world safety signals and informing go/no-go decisions, dosing, and monitoring.	1,656	Classification	[37]
ClinTox	Uses the ClinTox benchmark to profile clinical safety, contrasting molecules with adverse clinical signals against approved exemplars, supporting judgments about translational viability.	1,682	Classification	[38]
Crustacean	Assesses acute and chronic toxicity to crustaceans (e.g., Daphnia, shrimp); outputs shape environmental and ecological safety profiles and influence regulatory review, labeling, and use restrictions	791	Classification	[39]

Developmental_and_Reproductive_Toxicity_Developmental_Toxicity (DRTDT)	Identifies compounds with developmental toxicity risk (e.g., teratogenicity or impaired embryonic development), guiding pregnancy labeling, contraception requirements in trials, and population eligibility	257	Classification	[40]
Dili	Flags compounds associated with drug-induced liver injury, including signals such as transaminase elevations, cholestasis, or liver failure; outcomes directly inform go/no-go decisions, dose selection, and clinical monitoring priorities.	1,038	Classification	[41]
Eye_Corrosion	Assesses potential for irreversible ocular tissue damage (corneal/iridal burns) and supports severe eye hazard classification and regulatory labeling, shaping formulation choices and use conditions.	2,730	Classification	[42]
Eye_Irritation	Assesses reversible ocular irritation (e.g., conjunctival redness, corneal opacity), informing eye hazard classification and labeling, with implications for exposure limits and risk communication.	6,168	Classification	[42]
H_ht	Identifies compounds linked to human hepatotoxicity (from human evidence aggregates), complementing DILI and guiding dose ranges, safety monitoring endpoints, and population eligibility.	1,276	Classification	[43]
Hepatotoxicity	Flags compounds associated with hepatotoxicity, covering signals such as cytolysis, cholestasis, and mitochondrial dysfunction; outcomes guide go/no-go decisions, dose ranges, and safety monitoring focus.	3,222	Classification	[44]
Micronucleus_Tox	Assesses micronucleus formation as an indicator of clastogenic or aneugenic potential; positive results signal possible carcinogenic risk and inform structural optimization and regulatory assessment.	752	Classification	[45]
NR_AhR	Evaluates activation or inhibition at the aryl hydrocarbon receptor (AhR), which regulates xenobiotic metabolism genes and impacts immune and developmental pathways; aberrant activity may indicate enzyme induction–driven interactions and related toxicity risks.	7,887	Classification	[46]
NR_AR	The androgen receptor (AR) is a nuclear	8,600	Classification	[46]

	receptor that mediates androgen signaling and contributes to metabolic homeostasis, reproduction, and development; dysregulated AR activity is implicated in prostate-related diseases. This task assesses compound activity at the AR pathway (agonism or antagonism) to flag potential endocrine disruption.			
NR_AR_LBD	NR_AR_LBD targets direct binding or modulation at the AR ligand-binding domain (LBD), capturing interactions within the receptor pocket and helping to differentiate ligand-level agonist/antagonist risk.	7,101	Classification	47
NR_Aromatase	Endocrine disrupting chemicals (EDCs) interfere with the biosynthesis and normal functions of steroid hormones including estrogen and androgen in the body. Aromatase catalyzes the conversion of androgen to estrogen and plays a key role in maintaining the androgen and estrogen balance in many of the EDC-sensitive organs.	6,761	Classification	48
NR_ER	The estrogen receptor (ER; ERα and ERβ) is a nuclear receptor involved in development, metabolic homeostasis, and reproduction. This task measures compound activity on ER signaling (agonism or antagonism) to identify potential estrogenic or anti-estrogenic effects.	7,370	Classification	49
NR_ER_LBD	NR_ER_LBD focuses on direct binding/modulation at the ER ligand-binding domain, providing readouts of affinity and structure–activity at the receptor pocket to elucidate ligand-driven endocrine disruption risk.	8,097	Classification	46
NR_GR	The glucocorticoid receptor GR (NR3C1) is a nuclear receptor that, upon ligand binding, translocates to the nucleus and binds glucocorticoid response elements to regulate stress responses, inflammation and immunity, and glucose and lipid metabolism. This task evaluates compound activity on the GR pathway (agonism or antagonism) to flag potential endocrine disruption, immunomodulatory effects, and metabolism-related liabilities.	7,133	Classification	50
NR_PPAR_gamm	The peroxisome proliferator-activated receptors	7,572	Classification	51

a	(PPARs) are lipid-activated transcription factors of the nuclear receptor superfamily with three distinct subtypes namely PPAR alpha, PPAR delta (also called PPAR beta) and PPAR gamma (PPARg). All these subtypes heterodimerize with Retinoid X receptor (RXR) and these heterodimers regulate transcription of various genes. PPAR-gamma receptor (glitazone receptor) is involved in the regulation of glucose and lipid metabolism.			
NR_TR	The thyroid hormone receptor TR (THRA/THRB) is a nuclear receptor that binds triiodothyronine (T3) to regulate gene transcription, impacting neurodevelopment, skeletal growth, cardiovascular function, and energy metabolism. This task evaluates compound activity on the TR pathway (agonism, antagonism, or ligand-binding-domain interactions) to flag potential endocrine disruption and associated neurodevelopmental and metabolic liabilities.	6,320	Classification	[52]
Respiratory_Tox	Respiratory toxicity assesses whether a compound injures the respiratory system, ranging from subtle functional impairment to clinically significant lung injury. Because early signs can be nonspecific, positive findings signal a meaningful safety risk that has led to drug withdrawals and warrants careful interpretation of exposure margins.	4,472	Classification	[53]
RLM	RLM (rat liver microsomes) measures microsomal metabolic stability and intrinsic clearance, typically reported as t1/2 or Clint. Results indicate propensity for hepatic clearance and first-pass metabolism in rat and help contextualize species differences when compared with human liver microsomes.	6,585	Classification	[54]
SR_ARE	SR_ARE captures activation of the Nrf2/antioxidant response element pathway, a canonical marker of cellular response to oxidative or electrophilic stress. Activation suggests redox stress potential and engagement of cytoprotective gene programs.	6,536	Classification	[55]
SR_ATAD5	SR_ATAD5 reports induction of ATAD5 (ELG1), a replication-coupled genome stability	8,202	Classification	[46]

	factor. Activation indicates DNA replication stress or DNA damage response and flags potential genotoxic liability.			
SR_HSE	SR_HSE monitors heat shock element–driven transcription as a readout of proteotoxic stress and unfolded protein response. Activation denotes disruption of protein homeostasis that can precede broader cellular injury.	1,774	Classification	46
SR_MMP	SR_MMP measures changes in mitochondrial membrane potential, a key indicator of mitochondrial function and cellular energy status. Loss of membrane potential suggests impaired oxidative phosphorylation and risk of apoptosis.	7,082	Classification	56
SR_P53	SR_P53 detects activation of the p53 pathway, which integrates cellular responses to DNA damage and stress. Increased activity indicates engagement of repair, cell-cycle arrest, or apoptosis programs consistent with genotoxic or other stressors.	8,213	Classification	57
BioconcF	A high BioconcF indicates propensity to accumulate in organisms and potentially magnify through food chains, signaling environmental persistence and ecological risk. For drugs and chemicals, this ties to environmental exposure, long-term residues, regulatory flags, and potential indirect human exposure considerations.	813	Regression	58
FDAMDD	The FDA Maximum Recommended Daily Dose (MRDD) is a human-referenced exposure benchmark—the highest daily dose recommended for clinical use—commonly used to contextualize safety margins and approximate a tolerability threshold in humans.	1,200	Regression	59
LC50DM	The 48-hour Daphnia magna LC50 is the concentration of a test chemical in water (mg/L) that causes 50% mortality in Daphnia magna after 48 hours, serving as a standard metric of acute aquatic toxicity.	337	Regression	60
Pyriformis	Tetrahymena pyriformis toxicity (IGC50): the aqueous concentration that causes 50% growth inhibition of the protozoan T. pyriformis (typically at ~40 h). It is a standard proxy for baseline narcosis–type aquatic toxicity; lower	1,484	Regression	61

	IGC50 indicates higher intrinsic cytotoxicity and greater environmental hazard.			
Rat_Chronic	Rat chronic toxicity summarizing long-term systemic effects under repeated dosing, commonly expressed as LOAEL/NOAEL from chronic oral studies. It reflects cumulative toxicity that guides safe starting dose selection, exposure limits, and risks associated with prolonged use.	378	Regression	[62]
Skin_Permeability	Skin permeability (often reported as log Kp) quantifies a compound's ability to cross the stratum corneum, shaping dermal absorption, systemic exposure from topical or incidental contact, and the feasibility of transdermal delivery.	186	Regression	[63]

**Physicochemical Properties**				
PAINS	PAINS denote substructures prone to assay interference and false positives across diverse screens, degrading hit quality, reproducibility, and the efficiency of lead optimization.	1,732	Classification	[64]
Basic_pKa	Basic pKa quantifies the propensity of a molecule's most basic site to protonate, thereby determining the charge state at physiological pH and influencing solubility, permeability, distribution, protein binding, clearance, and salt/formulation selection.	4,347	Regression	[65]
ESOL	ESOL (aqueous solubility) describes a compound's solubility in water, directly constraining oral absorption and systemic exposure and impacting formulation feasibility and the robustness of in-vitro assays.	1,322	Regression	[66]
logVP	LogVP (log vapor pressure) captures a compound's volatility and tendency to partition into the air phase, relevant to environmental fate, inhalation exposure assessment, and storage/process safety.	3,077	Regression	[67]
pkb	pKb complements the conjugate acid's pKa in defining base strength, thereby governing protonation and charge state and affecting	4,347	Regression	[68]

	solubility, membrane permeability, and tissue distribution.

2. The data distribution and scoring metrics for all bioactivity tasks

Table S2. Data distribution and scoring metrics for all bioactivity tasks

Task CHEMBL ID	Target name	Type	Dataset Size	Task CHEMBL ID	Target name	Type	Dataset Size
CHEMBL _1871	Androgen Receptor (AR)	K_i	659	CHEMBL _264	Histamine H3 receptor (HRH3)	K_i	2,862
CHEMBL _218	Cannabinoid receptor 1 (CB1)	EC_{50}	1,031	CHEMBL _2835	Janus kinase 1 (JAK1)	K_i	615
CHEMBL _244	Coagulation factor X (FX)	K_i	3,097	CHEMBL _2971	Janus kinase 2 (JAK2)	K_i	976
CHEMBL _236	Delta opioid receptor (DOR)	K_i	2,598	CHEMBL _237	Kappa opioid receptor (KOR) agonism	EC_{50}	955
CHEMBL _234	Dopamine D3 receptor (D3R)	K_i	3,657	CHEMBL _237	Kappa opioid receptor (KOR) inhibition	K_i	2,602
CHEMBL _219	Dopamine D4 receptor (D4R)	K_i	1,859	CHEMBL _233	μ-opioid receptor (MOR)	K_i	3,142
CHEMBL _238	Dopamine transporter (DAT)	K_i	1,052	CHEMBL _4792	Orexin receptor 2 (OX2R)	K_i	1,471
CHEMBL _4203	Dual specificity protein kinase CLK4	K_i	731	CHEMBL _239	Peroxisome proliferator-activated receptor alpha (PPARα)	EC_{50}	1,721
CHEMBL _2047	Farnesoid X receptor (FXR)	EC_{50}	631	CHEMBL _3979	Peroxisome proliferator-activated receptor delta (PPARδ)	EC_{50}	1,125
CHEMBL _4616	Ghrelin receptor (GHSR)	EC_{50}	682	CHEMBL _235	Peroxisome proliferator-activated receptor gamma (PPARγ)	EC_{50}	2,349
CHEMBL _2034	Glucocorticoid receptor (GR)	K_i	750	CHEMBL _4005	PI3-kinase p110-alpha subunit (PIK3CA)	K_i	960
CHEMBL _262	Glycogen synthase	K_i	856	CHEMBL _2147	Serine/threonine-protein kinase	K_i	1,456

	kinase-3 beta (GSK3)				PIM1		
CHEMBL _231	Histamine H1 receptor (HRH1)	K_i	973	CHEMBL _214	Serotonin 1a receptor (5-HT1A)	K_i	3,317
CHEMBL _264	Histamine H3 receptor (HRH3)	K_i	2,862	CHEMBL _228	Serotonin transporter (SERT)	K_i	1,704
CHEMBL _204	Thrombin	K_i	2,754	CHEMBL _1862	Tyrosine-protein kinase ABL1	K_i	794
CHEMBL _2835	Janus kinase 1 (JAK1)	K_i	615	CHEMBL _287	Sigma opioid receptor (SOR)	K_i	2,328

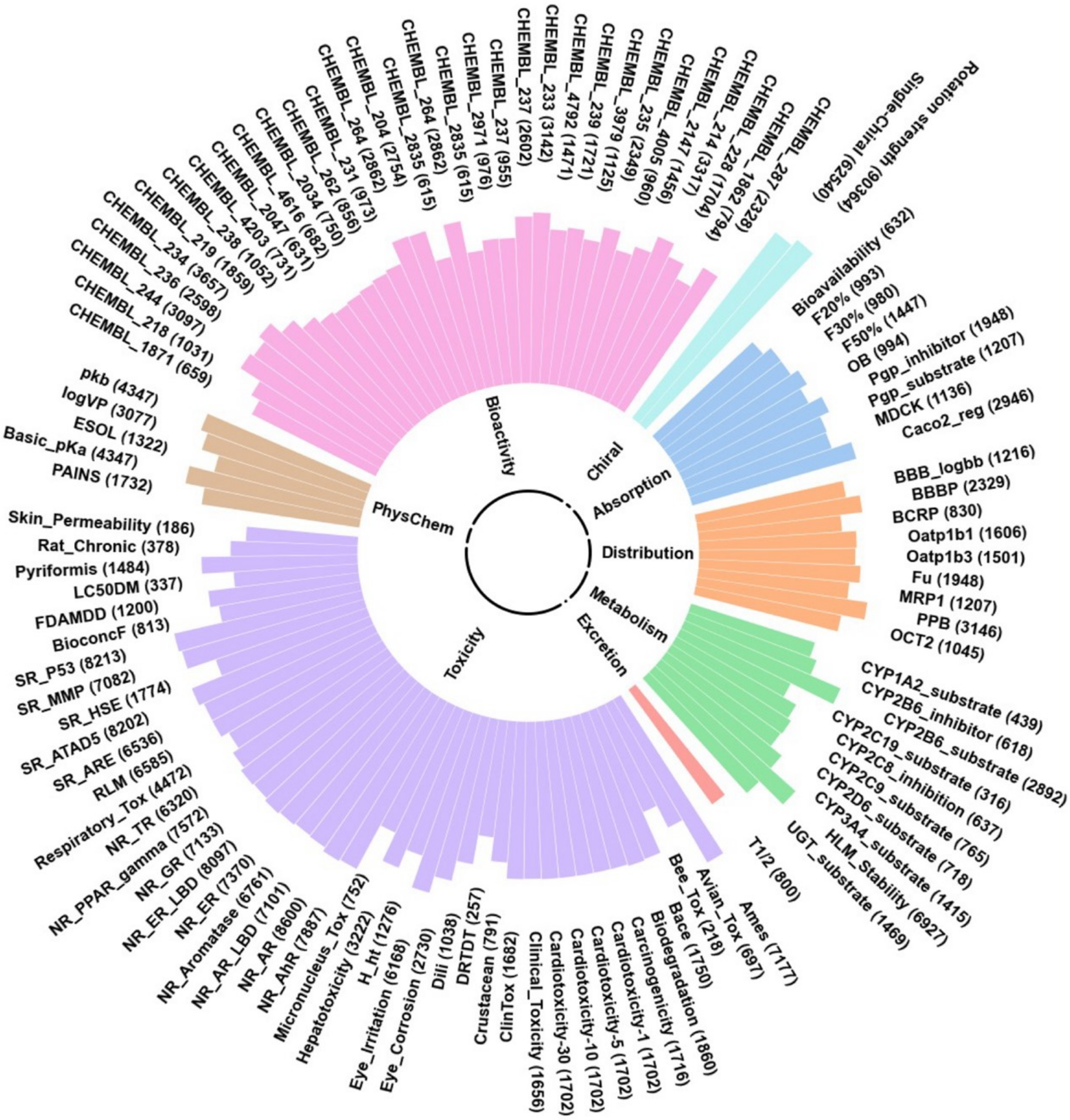

Figure S1. Task distribution of the large-scale molecular property dataset across eight categories: Absorption, Distribution, Metabolism, Excretion, Toxicity, Physicochemical, Bioactivity, and Chirality.

3. The performance of CAMF comparing baseline models

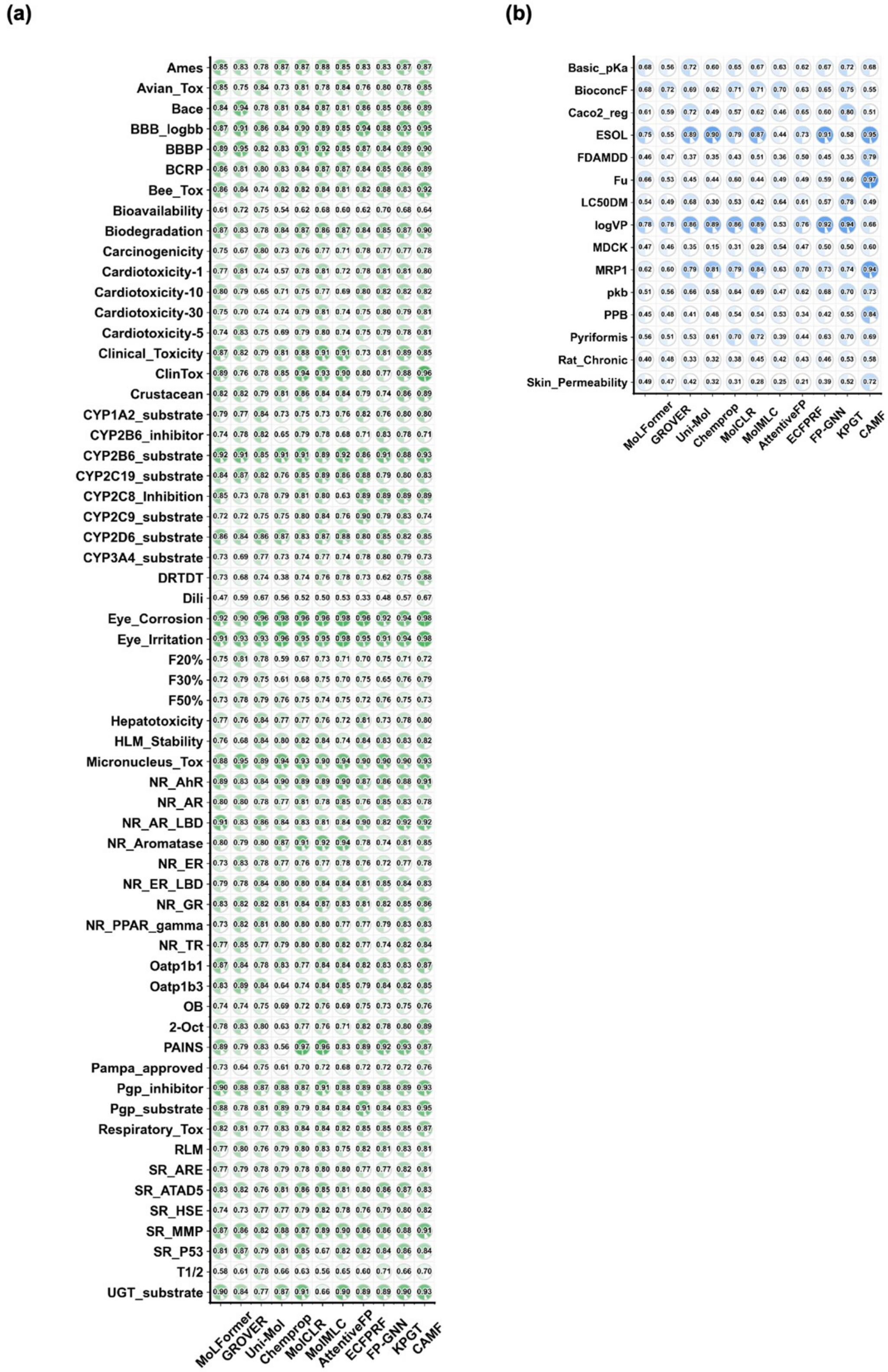

Figure S2. Heatmaps comparing model performance. Panel a shows AUC across multiple classification tasks. Panel b shows R2 across multiple regression tasks.

4. The performance of CAMF comparing unimodal features

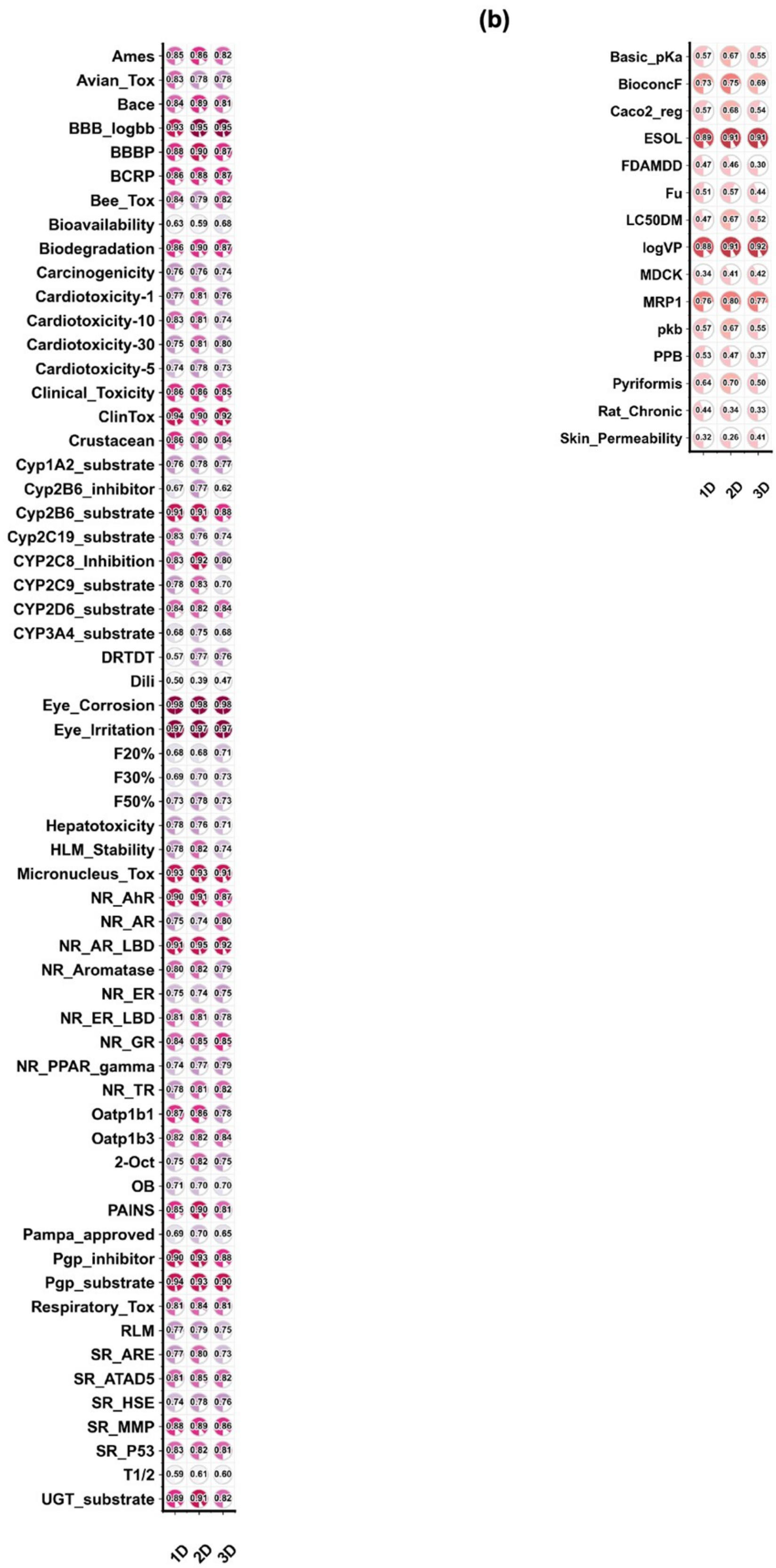

Figure S3. Heatmaps comparing model performance. a shows AUC classification tasks. b shows R^2 regression tasks.

5. The performance of CAMF comparing baseline in activity cliff

Table S3. Performance (R^2) of 10 methods on 30 binding potency prediction tasks in activity cliff.

Task	CHEMPROT	MolCLR	MolMCL	AttentiveFP	ECFP_RF	FP-GNN	KPGT	CAMF_emb	CAMF_dec	CAMF
CHEMBL1862_Ki	0.53	0.64	**0.81**	0.52	0.66	0.47	0.73	0.75	0.75	0.78
CHEMBL1871 Ki	0.41	0.49	0.61	0.41	0.59	0.44	0.64	0.59	0.65	**0.66**
CHEMBL2034 Ki	0.2	0.32	0.52	0.3	0.43	0.51	0.53	0.41	0.54	**0.56**
CHEMBL204 Ki	0.49	0.61	**0.81**	0.45	0.7	0.77	0.77	0.70	0.75	0.76
CHEMBL2047 EC50	0.24	0.51	0.31	0.19	0.54	0.28	**0.61**	0.48	0.56	0.57
CHEMBL214_Ki	0.47	0.6	0.62	0.3	0.58	0.61	**0.64**	0.56	0.61	**0.64**
CHEMBL2147 Ki	0.69	0.83	0.79	0.73	0.85	0.52	0.85	0.88	0.89	**0.91**
CHEMBL218 EC50	0.19	0.42	0.53	0.18	0.49	**0.75**	0.60	0.45	0.53	0.56
CHEMBL219 Ki	0.24	0.37	0.39	0.18	0.51	0.58	0.60	0.44	0.54	**0.61**
CHEMBL228 Ki	0.44	0.56	0.58	0.34	0.61	0.59	0.67	0.55	0.68	**0.68**
CHEMBL231_Ki	0.45	0.63	0.74	0.43	0.57	0.72	0.67	0.70	0.72	**0.75**
CHEMBL233 Ki	0.44	0.5	**0.70**	0.38	0.58	0.63	0.64	0.58	0.66	0.66
CHEMBL234 Ki	0.45	0.52	0.68	0.37	0.62	**0.70**	0.67	0.56	0.64	0.65
CHEMBL235 EC50	0.4	0.55	0.65	0.25	0.6	0.54	0.6	0.61	0.65	**0.66**
CHEMBL236 Ki	0.51	0.6	0.71	0.47	0.68	0.68	0.69	0.67	0.71	**0.72**
CHEMBL237_EC50	0.32	0.6	0.71	0.37	0.68	0.65	0.69	0.63	0.71	**0.71**
CHEMBL237 Ki	0.5	0.59	0.72	0.45	0.66	0.66	0.7	0.68	0.73	**0.74**
CHEMBL238 Ki	0.38	0.65	0.62	0.27	0.68	0.69	0.72	0.64	0.72	**0.74**
CHEMBL239 EC50	0.31	0.44	0.46	0.23	0.57	0.57	**0.61**	0.53	0.58	0.57
CHEMBL244 Ki	0.57	0.68	**0.80**	0.48	0.75	0.44	0.79	0.74	0.80	**0.80**
CHEMBL262_Ki	0.25	0.4	0.58	0.2	0.53	0.48	**0.59**	0.32	0.50	0.55
CHEMBL264 Ki	0.41	0.56	0.69	0.4	0.59	0.42	0.68	0.62	0.68	**0.70**
CHEMBL2835 Ki	0.46	0.76	0.81	0.75	0.8	0.42	0.77	0.84	0.83	**0.85**
CHEMBL287 Ki	0.29	0.36	**0.88**	0.35	0.42	0.4	0.59	0.53	0.61	0.64
CHEMBL2971 Ki	0.48	0.71	**0.84**	0.64	0.76	0.46	0.77	0.78	0.78	0.79
CHEMBL3979_EC50	0.43	0.57	0.56	0.38	0.63	**0.7**	0.69	0.59	0.68	0.67
CHEMBL4005 Ki	0.34	0.53	0.54	0.42	0.61	0.33	0.61	0.61	0.64	**0.65**
CHEMBL4203 Ki	0.12	0.17	0.38	0.17	0.27	**0.44**	0.3	0.25	0.29	0.31
CHEMBL4616 EC50	0.17	0.3	**0.44**	0.16	0.36	0.31	**0.44**	0.29	0.37	0.39
CHEMBL4792 Ki	0.36	0.56	0.49	0.36	0.6	0.6	0.68	0.60	0.68	**0.69**
Average	0.38	0.53	0.63	0.37	0.60	0.55	0.65	0.59	0.65	**0.665**

6. Mechanistic and translational case studies support practical utility

Table S4. The evaluation of Regorafenib, Fialuridine, and Obeticholic across three tools of cardiotoxicity of Hepatotoxicity

Tool	Drug	Structures	Hepatotoxicity
CAMF			Alert
admetSAR 3.0	Obeticholic acid	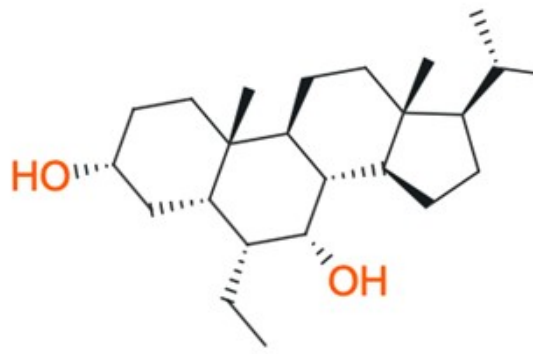	Alert
ADMETlab 3.0			Non-alert
CAMF			Alert
admetSAR 3.0	Regorafenib	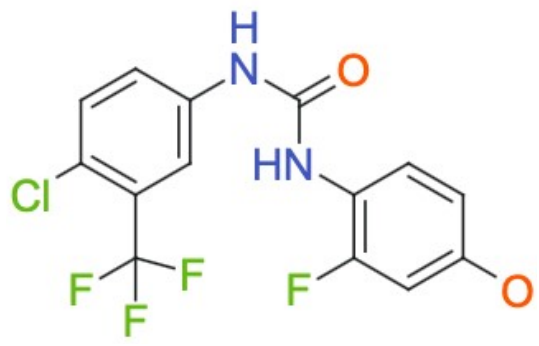	Alert
ADMETlab 3.0			Alert
CAMF			Alert
admetSAR 3.0	Fialuridine	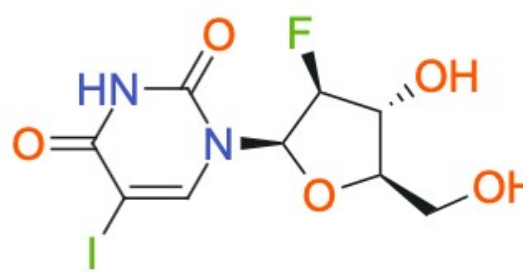	Alert
ADMETlab 3.0			Alert

References

1. Cabrera-Pérez MÁ., Pham-The H. Computational modeling of human oral bioavailability: what will be next? *Expert Opin Drug Discov* 2018;**13**(6):509–21.
2. Agoram B., Woltosz WS., Bolger MB. Predicting the impact of physiological and biochemical processes on oral drug bioavailability. *Adv Drug Deliv Rev* 2001;**50**:S41–67.
3. Falcón-Cano G., Molina C., Cabrera-Pérez MÁ. ADME Prediction with KNIME: Development and Validation of a Publicly Available Workflow for the Prediction of Human Oral Bioavailability. *J Chem Inf Model* 2020;**60**(6):2660–7. Doi: 10.1021/acs.jcim.0c00019.
4. Veber DF., Johnson SR., Cheng H-Y., Smith BR., Ward KW., Kopple KD. Molecular Properties That Influence the Oral Bioavailability of Drug Candidates. *J Med Chem* 2002;**45**(12):2615–23. Doi: 10.1021/jm020017n.
5. Varma MV., Ashokraj Y., Dey CS., Panchagnula R. P-glycoprotein inhibitors and their screening: a perspective from bioavailability enhancement. *Pharmacol Res* 2003;**48**(4):347–59.
6. Didziapetris R., Japertas P., Avdeef A., Petrauskas A. Classification analysis of P-glycoprotein substrate specificity. *J Drug Target* 2003;**11**(7):391–406.
7. Irvine JD., Takahashi L., Lockhart K., Cheong J., Tolan JW., Selick H., et al. MDCK (Madin–Darby canine kidney) cells: a tool for membrane permeability screening. *J Pharm Sci* 1999;**88**(1):28–33.
8. Wang N-N., Dong J., Deng Y-H., Zhu M-F., Wen M., Yao Z-J., et al. ADME properties evaluation in drug discovery: prediction of Caco-2 cell permeability using a combination of NSGA-II and boosting. *J Chem Inf Model* 2016;**56**(4):763–73.
9. Kunwittaya S., Nantasenamat C., Treeratanapiboon L., Srisarin A., Isarankura-Na-Ayudhya C., Prachayasittikul V. Influence of logBB cut-off on the prediction of blood-brain barrier permeability. *Biomed Appl Technol J* 2013;**1**:16–34.
10. Tong X., Wang D., Ding X., Tan X., Ren Q., Chen G., et al. Blood–brain barrier penetration prediction enhanced by uncertainty estimation. *J Cheminformatics* 2022;**14**(1):44.
11. Mao Q., Unadkat JD. Role of the breast cancer resistance protein (BCRP/ABCG2) in drug transport—an update. *AAPS J* 2015;**17**(1):65–82.
12. Kalliokoski A., Niemi M. Impact of OATP transporters on pharmacokinetics. *Br J Pharmacol* 2009;**158**(3):693–705.
13. Shitara Y. Clinical importance of OATP1B1 and OATP1B3 in drug–drug interactions. *Drug Metab Pharmacokinet* 2011;**26**(3):220–7.
14. Al-Qassabi J., Tan SPF., Phonboon P., Galetin A., Rostami-Hodjegan A., Scotcher D. Facing the facts of altered plasma protein binding: do current models correctly predict changes in fraction unbound in special populations? *J Pharm Sci* 2024;**113**(6):1664–73.
15. Bakos É., Homolya L. Portrait of multifaceted transporter, the multidrug resistance-associated protein 1 (MRP1/ABCC1). *Pflüg Arch-Eur J Physiol* 2007;**453**(5):621–41.
16. Di L. An update on the importance of plasma protein binding in drug discovery and development. *Expert Opin Drug Discov* 2021;**16**(12):1453–65.
17. Suo Y., Wright NJ., Guterres H., Fedor JG., Butay KJ., Borgnia MJ., et al. Molecular basis of polyspecific drug and xenobiotic recognition by OCT1 and OCT2. *Nat Struct Mol Biol* 2023;**30**(7):1001–11. Doi: 10.1038/s41594-023-01017-4.
18. Zhou S-F., Yang L-P., Zhou Z-W., Liu Y-H., Chan E. Insights into the substrate specificity,

inhibitors, regulation, and polymorphisms and the clinical impact of human cytochrome P450 1A2. *AAPS J* 2009;**11**(3):481–94.

19. Turpeinen M., Raunio H., Pelkonen O. The functional role of CYP2B6 in human drug metabolism: substrates and inhibitors in vitro, in vivo and in silico. *Curr Drug Metab* 2006;**7**(7):705–14.
20. EKINS S., WRIGHTON SA. The role of CYP2B6 in human xenobiotic metabolism. *Drug Metab Rev* 1999;**31**(3):719–54.
21. Foti RS., Wahlstrom JL. CYP2C19 inhibition: the impact of substrate probe selection on in vitro inhibition profiles. *Drug Metab Dispos* 2008;**36**(3):523–8.
22. VandenBrink BM., Foti RS., Rock DA., Wienkers LC., Wahlstrom JL. Evaluation of CYP2C8 inhibition in vitro: utility of montelukast as a selective CYP2C8 probe substrate. *Drug Metab Dispos* 2011;**39**(9):1546–54.
23. Maekawa K., Harakawa N., Sugiyama E., Tohkin M., Kim S-R., Kaniwa N., et al. Substrate-dependent functional alterations of seven CYP2C9 variants found in Japanese subjects. *Drug Metab Dispos* 2009;**37**(9):1895–903.
24. Wang A., Stout CD., Zhang Q., Johnson EF. Contributions of ionic interactions and protein dynamics to cytochrome P450 2D6 (CYP2D6) substrate and inhibitor binding. *J Biol Chem* 2015;**290**(8):5092–104.
25. Galetin A., Ito K., Hallifax D., Houston JB. CYP3A4 substrate selection and substitution in the prediction of potential drug-drug interactions. *J Pharmacol Exp Ther* 2005;**314**(1):180–90.
26. Liu R., Schyman P., Wallqvist A. Critically assessing the predictive power of QSAR models for human liver microsomal stability. *J Chem Inf Model* 2015;**55**(8):1566–75.
27. Huang M., Lou C., Wu Z., Li W., Lee PW., Tang Y., et al. In silico prediction of UGT-mediated metabolism in drug-like molecules via graph neural network. *J Cheminformatics* 2022;**14**(1):46.
28. Swanson K., Walther P., Leitz J., Mukherjee S., Wu JC., Shivnaraine RV., et al. ADMET-AI: a machine learning ADMET platform for evaluation of large-scale chemical libraries. *Bioinformatics* 2024;**40**(7):btae416.
29. Guan L., Yang H., Cai Y., Sun L., Di P., Li W., et al. ADMET-score–a comprehensive scoring function for evaluation of chemical drug-likeness. *Medchemcomm* 2019;**10**(1):148–57.
30. Newsted JL., Jones PD., Coady K., Giesy JP. Avian toxicity reference values for perfluorooctane sulfonate. *Environ Sci Technol* 2005;**39**(23):9357–62.
31. Adamczyk J., Poziemski J., Siedlecki P. ApisTox: a new benchmark dataset for the classification of small molecules toxicity on honey bees. *Sci Data* 2025;**12**(1):5.
32. Wu Z., Ramsundar B., Feinberg EN., Gomes J., Geniesse C., Pappu AS., et al. MoleculeNet: a benchmark for molecular machine learning. *Chem Sci* 2018;**9**(2):513–30.
33. He Y., Deng X., Jiang L., Hao L., Shi Y., Lyu M., et al. Current advances, challenges and strategies for enhancing the biodegradation of plastic waste. *Sci Total Environ* 2024;**906**:167850.
34. Cogliano V., Baan R., Straif K., Grosse Y., Secretan B., El Ghissassi F. Carcinogenicity of human papillomaviruses. *Lancet Oncol* 2005;**6**(4):204.
35. Braña I., Zamora E., Tabernero J. Cardiotoxicity. *Side Eff Med Cancer Ther Prev Treat* 2012:483–530.
36. Ewer MS., Ewer SM. Cardiotoxicity of anticancer treatments. *Nat Rev Cardiol*

2015;**12**(9):547–58.
37. Bai C., Wu L., Li R., Cao Y., He S., Bo X. Machine Learning-Enabled Drug-Induced Toxicity Prediction. *Adv Sci* 2025;**12**(16):2413405.
38. Hill SL., Thomas SH. Clinical toxicology of newer recreational drugs. *Clin Toxicol* 2011;**49**(8):705–19.
39. LeBlanc GA. Crustacean endocrine toxicology: a review. *Ecotoxicology* 2007;**16**(1):61–81.
40. Tan H., Wu J., Zhang R., Zhang C., Li W., Chen Q., et al. Development, validation, and application of a human reproductive toxicity prediction model based on adverse outcome pathway. *Environ Sci Technol* 2022;**56**(17):12391–403.
41. Andrade RJ., Chalasani N., Björnsson ES., Suzuki A., Kullak-Ublick GA., Watkins PB., et al. Drug-induced liver injury. *Nat Rev Dis Primer* 2019;**5**(1):58.
42. Clippinger AJ., Raabe HA., Allen DG., Choksi NY., van der Zalm AJ., Kleinstreuer NC., et al. Human-relevant approaches to assess eye corrosion/irritation potential of agrochemical formulations. *Cutan Ocul Toxicol* 2021;**40**(2):145–67.
43. Fu L., Shi S., Yi J., Wang N., He Y., Wu Z., et al. ADMETlab 3.0: an updated comprehensive online ADMET prediction platform enhanced with broader coverage, improved performance, API functionality and decision support. *Nucleic Acids Res* 2024;**52**(W1):W422–31. Doi: 10.1093/nar/gkae236.
44. Lee WM. Drug-induced hepatotoxicity. *N Engl J Med* 2003;**349**(5):474–85.
45. Hayashi M. The micronucleus test—most widely used in vivo genotoxicity test—. *Genes Environ* 2016;**38**(1):18.
46. Banerjee P., Eckert AO., Schrey AK., Preissner R. ProTox-II: a webserver for the prediction of toxicity of chemicals. *Nucleic Acids Res* 2018;**46**(W1):W257–63.
47. de Sá AG., Long Y., Portelli S., Pires DE., Ascher DB. toxCSM: comprehensive prediction of small molecule toxicity profiles. *Brief Bioinform* 2022;**23**(5):bbac337.
48. Du H., Cai Y., Yang H., Zhang H., Xue Y., Liu G., et al. In silico prediction of chemicals binding to aromatase with machine learning methods. *Chem Res Toxicol* 2017;**30**(5):1209–18.
49. Lee H-R., Kim T-H., Choi K-C. Functions and physiological roles of two types of estrogen receptors, ERα and ERβ, identified by estrogen receptor knockout mouse. *Lab Anim Res* 2012;**28**(2):71–6.
50. Cavasotto CN., Scardino V. Machine learning toxicity prediction: latest advances by toxicity end point. *ACS Omega* 2022;**7**(51):47536–46.
51. Jiang J., Wang R., Wei G-W. GGL-Tox: geometric graph learning for toxicity prediction. *J Chem Inf Model* 2021;**61**(4):1691–700.
52. Flamant F., Baxter JD., Forrest D., Refetoff S., Samuels H., Scanlan TS., et al. International Union of Pharmacology. LIX. The pharmacology and classification of the nuclear receptor superfamily: thyroid hormone receptors. *Pharmacol Rev* 2006;**58**(4):705–11.
53. Wang Z., Zhao P., Zhang X., Xu X., Li W., Liu G., et al. In silico prediction of chemical respiratory toxicity via machine learning. *Comput Toxicol* 2021;**18**:100155.
54. Amer SM., Kadi AA., Darwish HW., Attwa MW. Liquid chromatography tandem mass spectrometry method for the quantification of vandetanib in human plasma and rat liver microsomes matrices: metabolic stability investigation. *Chem Cent J* 2017;**11**(1):45.
55. Chen X-L., Kunsch C. Induction of cytoprotective genes through Nrf2/antioxidant response element pathway: a new therapeutic approach for the treatment of inflammatory diseases. *Curr*

Pharm Des 2004;**10**(8):879–91.

56. Yao Q., Shen Z., Wang Y., Dou D. Property-Aware Relation Networks for Few-Shot Molecular Property Prediction. *IEEE Trans Pattern Anal Mach Intell* 2024;**46**(8):5413–29. Doi: 10.1109/TPAMI.2024.3368090.
57. Chithrananda S., Grand G., Ramsundar B. ChemBERTa: large-scale self-supervised pretraining for molecular property prediction. *ArXiv Prepr ArXiv201009885* 2020.
58. Han M., Joung JF., Jeong M., Choi DH., Park S. Generative Deep Learning-Based Efficient Design of Organic Molecules with Tailored Properties. *ACS Cent Sci* 2025;**11**(2):219–27. Doi: 10.1021/acscentsci.4c00656.
59. Herndon CM., Dankenbring DM. Patient perception and knowledge of acetaminophen in a large family medicine service. *J Pain Palliat Care Pharmacother* 2014;**28**(2):109–16.
60. Chen D., Gao K., Nguyen DD., Chen X., Jiang Y., Wei G-W., et al. Algebraic graph-assisted bidirectional transformers for molecular property prediction. *Nat Commun* 2021;**12**(1):3521.
61. Sauvant N., Pepin D., Piccinni E. Tetrahymena pyriformis: a tool for toxicological studies. A review. *Chemosphere* 1999;**38**(7):1631–69.
62. Mazzatorta P., Estevez MD., Coulet M., Schilter B. Modeling oral rat chronic toxicity. *J Chem Inf Model* 2008;**48**(10):1949–54.
63. Potts RO., Guy RH. Predicting skin permeability. *Pharm Res* 1992;**9**(5):663–9.
64. Baell JB., Holloway GA. New substructure filters for removal of pan assay interference compounds (PAINS) from screening libraries and for their exclusion in bioassays. *J Med Chem* 2010;**53**(7):2719–40.
65. Rupp M., Korner R., V Tetko I. Predicting the pKa of small molecules. *Comb Chem High Throughput Screen* 2011;**14**(5):307–27.
66. Delaney JS. ESOL: estimating aqueous solubility directly from molecular structure. *J Chem Inf Comput Sci* 2004;**44**(3):1000–5.
67. Zang Q., Mansouri K., Williams AJ., Judson RS., Allen DG., Casey WM., et al. In silico prediction of physicochemical properties of environmental chemicals using molecular fingerprints and machine learning. *J Chem Inf Model* 2017;**57**(1):36–49.
68. Avdeef A. Physicochemical profiling (solubility, permeability and charge state). *Curr Top Med Chem* 2001;**1**(4):277–351.
